# Epigenetic modifier alpha-ketoglutarate modulates aberrant gene body methylation and hydroxymethylation marks in diabetic heart

**DOI:** 10.1186/s13072-023-00489-4

**Published:** 2023-04-27

**Authors:** Rohini Dhat, Dattatray Mongad, Sivarupa Raji, Silpa Arkat, Nitish R. Mahapatra, Nishant Singhal, Sandhya L. Sitasawad

**Affiliations:** 1 National Centre for Cell Science, NCCS Complex, S. P. Pune University, Ganeshkhind, Pune, Maharashtra 411007 India; 2 NCMR-National Centre for Cell Science (NCCS), Pune, Maharashtra 411007 India; 3 Department of Biotechnology, Bhupat and Jyoti Mehta School of Biosciences, Indian Institute of Technology Madras, Chennai, 600036 India; 4 Reginal Centre for Biotechnology, 3rd Milestone, Faridabad-Gurgaon Expressway, Faridabad Rd, Haryana, 121001 India

**Keywords:** Diabetic cardiomyopathy, Epigenetics, DNA methylation, DNA hydroxymethylation, 5mC, 5hmC, DNMT3B, MEDIP-seq, hMEDIP-seq, Alpha-ketoglutarate

## Abstract

**Background:**

Diabetic cardiomyopathy (DCM) is a leading cause of death in diabetic patients. Hyperglycemic myocardial microenvironment significantly alters chromatin architecture and the transcriptome, resulting in aberrant activation of signaling pathways in a diabetic heart. Epigenetic marks play vital roles in transcriptional reprogramming during the development of DCM. The current study is aimed to profile genome-wide DNA (hydroxy)methylation patterns in the hearts of control and streptozotocin (STZ)-induced diabetic rats and decipher the effect of modulation of DNA methylation by alpha-ketoglutarate (AKG), a TET enzyme cofactor, on the progression of DCM.

**Methods:**

Diabetes was induced in male adult Wistar rats with an intraperitoneal injection of STZ. Diabetic and vehicle control animals were randomly divided into groups with/without AKG treatment. Cardiac function was monitored by performing cardiac catheterization. Global methylation (5mC) and hydroxymethylation (5hmC) patterns were mapped in the Left ventricular tissue of control and diabetic rats with the help of an enrichment-based (h)MEDIP-sequencing technique by using antibodies specific for 5mC and 5hmC. Sequencing data were validated by performing (h)MEDIP-qPCR analysis at the gene-specific level, and gene expression was analyzed by qPCR. The mRNA and protein expression of enzymes involved in the DNA methylation and demethylation cycle were analyzed by qPCR and western blotting. Global 5mC and 5hmC levels were also assessed in high glucose-treated DNMT3B knockdown H9c2 cells.

**Results:**

We found the increased expression of DNMT3B, MBD2, and MeCP2 with a concomitant accumulation of 5mC and 5hmC, specifically in gene body regions of diabetic rat hearts compared to the control. Calcium signaling was the most significantly affected pathway by cytosine modifications in the diabetic heart. Additionally, hypermethylated gene body regions were associated with Rap1, apelin, and phosphatidyl inositol signaling, while metabolic pathways were most affected by hyperhydroxymethylation. AKG supplementation in diabetic rats reversed aberrant methylation patterns and restored cardiac function. Hyperglycemia also increased 5mC and 5hmC levels in H9c2 cells, which was normalized by DNMT3B knockdown or AKG supplementation.

**Conclusion:**

This study demonstrates that reverting hyperglycemic damage to cardiac tissue might be possible by erasing adverse epigenetic signatures by supplementing epigenetic modulators such as AKG along with an existing antidiabetic treatment regimen.

**Supplementary Information:**

The online version contains supplementary material available at 10.1186/s13072-023-00489-4.

## Background

Diabetic cardiomyopathy (DCM) is the main cardiac complication and a leading cause of death in patients with diabetes due to heart failure [1]. Chronic hyperglycemia is associated with fibrosis-driven myocardial stiffness, cardiomyocyte hypertrophy, and altered substrate metabolism triggering structural and functional impairment in the cardiac tissue leading to DCM [2–5]. Glucose-induced progression of these cardiac pathologies is mediated through aberrant activation of the signaling pathways that are persuaded by changes in the cardiac transcriptome [6, 7]. The underlying molecular mechanisms involved in this transcriptional reprogramming are poorly understood. Epigenetics can explain how environmental stimuli such as hyperglycemia can trigger a pathological transcriptional response in cardiac cells. Epigenetic modifications such as microRNA, histone modifications, and DNA methylation have been previously associated with cardiac development and disease [9, 10]

DNA methylation is a stable and inheritable epigenetic modification. Chromatin undergoes a highly organized and regulated process of methylation and demethylation cycles. This is carried out by the association and coordination of multiple protein factors constituting the DNA methylation machinery and are termed writers, readers, and erasers of DNA methylation [11]. Writers include maintenance methyltransferase DNMT1 and de novo methyltransferase DNMT3A/DNMT3B, which catalyze the transfer of the methyl group from s-adenosylmethionine to cytosine at the 5ʹ position, forming 5ʹ methylcytosine (5mC), which is present mainly in CpG dinucleotides [12]. Readers are methyl-CpG binding domain proteins, including MBDs, MeCP2, and SETDBs*,* that recognize and bind to methylated DNA, thereby either inhibiting the binding of transcription factors or recruiting other transcriptional repressors, including histone deacetylases (HDACs) [13, 14]. Erasers catalyze the removal of these methylation marks known as ten eleven translocase enzymes (TET1, TET2, TET3), which are α-ketoglutarate (AKG)-dependent dioxygenases. The most stable intermediate product of the DNA demethylation process is 5ʹ hydroxymethyl cytosine (5hmC), which is further oxidized to 5-formylcytosine (5fC) and 5-carboxycytosine (5caC) and ultimately converted to cytosine with the help of thymine DNA glycosylase (TDG) and base excision repair mechanisms [15].

While the use of different epigenetic modulators, such as cytidine analogs 5-azacytidine and decitabine, as DNMT inhibitors for cancer treatment, is well documented, the therapeutic potential of these modulators is limited due to their lower stability and cytotoxicity. Additional side effects from treatment with these drugs were also reported in cancer patients with other comorbidities, such as T2D or cardiac disorders [16–19]. Moreover, the use of non-nucleoside analogs such as hydralazine and procainamide is also limited because of their specificity for DNMT1 and side effects such as autoimmune diseases [20]. Since most of these DNMT inhibitors work during DNA replication in mitotic cells, their use in postmitotic cardiac cells such as cardiomyocytes limits their use as an epigenetic modulator in cardiac complications, including DCM.α-ketoglutarate (AKG) is a Kreb’s cycle intermediate that plays an important role in cellular energy metabolism. It can also function as a demethylating agent by acting as a cofactor for TET enzymes in the presence of molecular oxygen and Fe(II) [21, 22]. AKG further helps to complete the oxidation of 5mC to cytosine by aiding in TDG function and the formation of the TET-TDG complex [23]. Previous reports have shown lower levels of AKG in cardiac mesenchymal cells isolated from T2D patients [23] and in the heart tissue of HFD-fed mice [24]. Thus, we aimed to investigate the effect of AKG supplementation as an epigenetic modulator in streptozotocin-induced DCM.

In the present study, we examined the effect of the cardiac hyperglycemic environment on genome-wide methylation and hydroxymethylation [(hydroxy)methylation] patterns and the expression of proteins involved in the DNA methylation and demethylation process in the left ventricles (LV) of STZ-induced diabetic rats. (Hydroxy)methylome analysis was performed by methylated DNA immunoprecipitation-sequencing (MEDIP-seq) and hydroxymethylated DNA immunoprecipitation-sequencing (hMEDIP-seq) analysis. Furthermore, we studied the therapeutic potential of the TET enzyme cofactor AKG in these diabetic animals. We have shown that diabetes not only increased global methylation but also hydroxymethylation in the hearts of STZ-induced diabetic rats. AKG supplementation restored the (hydroxy)methylation levels and cardiac function in diabetic animals.

## Results

### Diabetes changes the DNA methylation and hydroxymethylation landscape in heart tissue

**Fig. 1:**
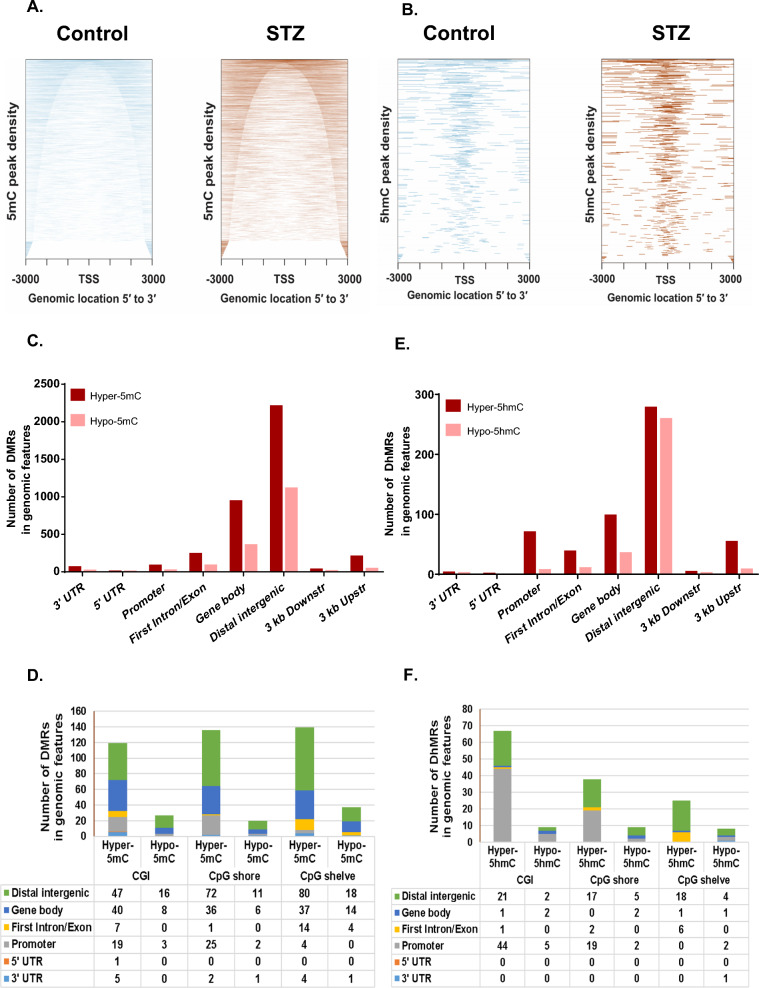
Genomic distribution of 5mC and 5hmC peaks in the LV tissue of control and diabetic rats. **A** Heat map of the genomic distribution of 5mC peaks relative to TSS. **B** Heat map of the genomic distribution of 5hmC peaks relative to TSS. **C**, **D** Distribution of DMRs in genomic elements that have increased (hyper-5mC) and decreased (hypo-5mC) methylation in LV of diabetic vs control rat, **C** overall distribution **D** according to CpG density. **E**, **F** Distribution of DhMRs in genomic elements with increased (hyper-5hmC) and decreased (hypo-5hmC) hydroxymethylation in the LV tissue of diabetic vs control rats. **E** Overall distribution **F** according to CpG density. The promoter regions are defined as 1 kb upstream of the TSS

MEDIP-seq and hMEDIP-seq analyses were performed to generate a genome-wide map of 5mC and 5hmC peaks in the left ventricles of control and diabetic animals. We found an increase in the peak density of 5mC and 5hmC in diabetic cardiac tissue. The heat map shows that the 5mC peaks were concentrated in the regions away from the TSS as opposed to 5hmC peaks, which were densely populated at and around the TSS site and promoter regions (Fig. 1A and B).

Differential analysis showed that the total number of DMRs (5367) was high compared to DhMRs (869) in diabetic hearts, which indicates that 5mC accumulation is a prominent epigenetic modification due to hyperglycemia. However, transcriptional changes associated with D(h)MRs depend on their genomic location, especially the presence of these DNA-modified bases around TSS, promoter, and gene body regions, which profoundly affects the transcriptional activity of the associated gene. Thus, we further mapped the distribution of D(h)MRs in different genomic elements. There was an overall increase in the 5mC and 5hmC marks in all the genomic elements. Most of the D(h)MRs were enriched in intergenic regions followed by gene body regions, including the first intron and exons and 3ʹ UTR (Fig. 1C and D). Moreover, we also investigated the distribution of D(h)MRs in the genomic elements according to the CpG densities. In this analysis, we found more enrichment of 5hmC peaks in CpG-dense regions, e.g., CpG islands and CpG shores located within promoter regions, compared to 5mC peaks (Fig. 1E and F). These results thus strongly suggest that chronic exposure of cardiac cells to high blood glucose levels results in the accumulation of 5mC and 5hmC marks predominantly in the transcriptional regulatory regions.

### KEGG pathway and GO enrichment analysis of D(h)MRs in diabetic left ventricles

**Fig. 2:**
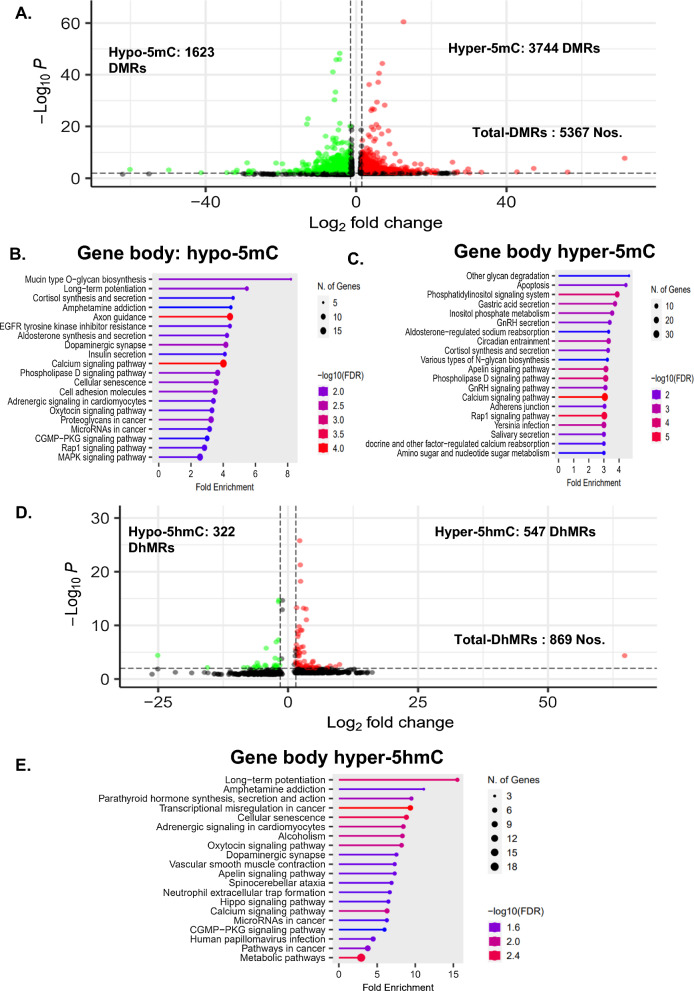
Volcano plot and pathway analysis of DMRs and DhMRs enriched in STZ vs control. **A** Volcano plot of the DMRs. Hyper-5mC regions (red) have 3744 peaks, hypo-5mC regions (green) have 1623 peaks, and a total of 5367 DMR peaks are shown. KEGG pathway analysis of the **B** hypermethylated gene body regions and **C** hypomethylated gene body regions. **D** Volcano plot of the DhMRs. Hyper-5hmC regions (red) had 547 peaks, and hypo-5hmC regions (green) had 322 peaks; a total of 869 DhMR peaks are shown. **E** KEGG pathway analysis of the hyperhydroxymethylated gene body regions. For KEGG pathway analysis on the y-axis enriched pathway and x-axis fold enrichment given, the size of the dot at the end of the bar represents the number of genes, and the color represents the level of significance

The volcano plot shows uneven distribution of D(h)MRs with an increased number of 5mC/5hmC peaks in diabetic LV tissue compared to the control (Fig. 2A and D). KEGG pathway analysis was performed for hyper(hydroxy)methylated and hypo(hydroxy)methylated D(h)MRs found in gene body regions, including exons/introns, UTRs, and promoters (Fig. 2B, C, E). KEGG pathway analysis of MEDIP-seq data revealed that the most significantly hypermethylated pathways included calcium signaling, Rap1 signaling, phospholipase D, apelin signaling, and phosphatidylinositol signaling. Moreover, calcium signaling pathway-related genes also appeared significantly in hypomethylated DMRs but the individual genes/regions included in this pathway were different for hyper-5mC and hypo-5mC DMRs. The list of the top 10 most significant pathway entries and the enrichment of the related genes for hypo-5mC and hyper-5mC are given in Additional file 1: Tables S1 and S2, respectively. Although there was no significant enrichment of any pathway for hypo-5hmC, we found a significant enrichment of DhMRs with hyper-5hmC for metabolic pathways followed by transcriptional misregulation in cancer, cellular senescence, calcium signaling, and adrenergic signaling in cardiomyocytes. The list of the top 10 most significant pathway entries and the enrichment of the related genes for hyper-5hmC are given in Additional file 1: Table S3. Overall, calcium signaling was the most affected pathway by 5mC and 5hmC accumulation. GO enrichment analysis also showed significant enrichment of genes related to signal transduction, calmodulin binding, MAPK signaling, and transcriptional regulation of gene expression (Additional file 1: Fig. S1). Overall, these results indicate the impact of DNA methylation on pathological signaling in diabetic cardiac complications.

### Validation of (h)MEDIP-seq data and functional relevance of 5mC/5hmC to gene expression

**Fig. 3:**
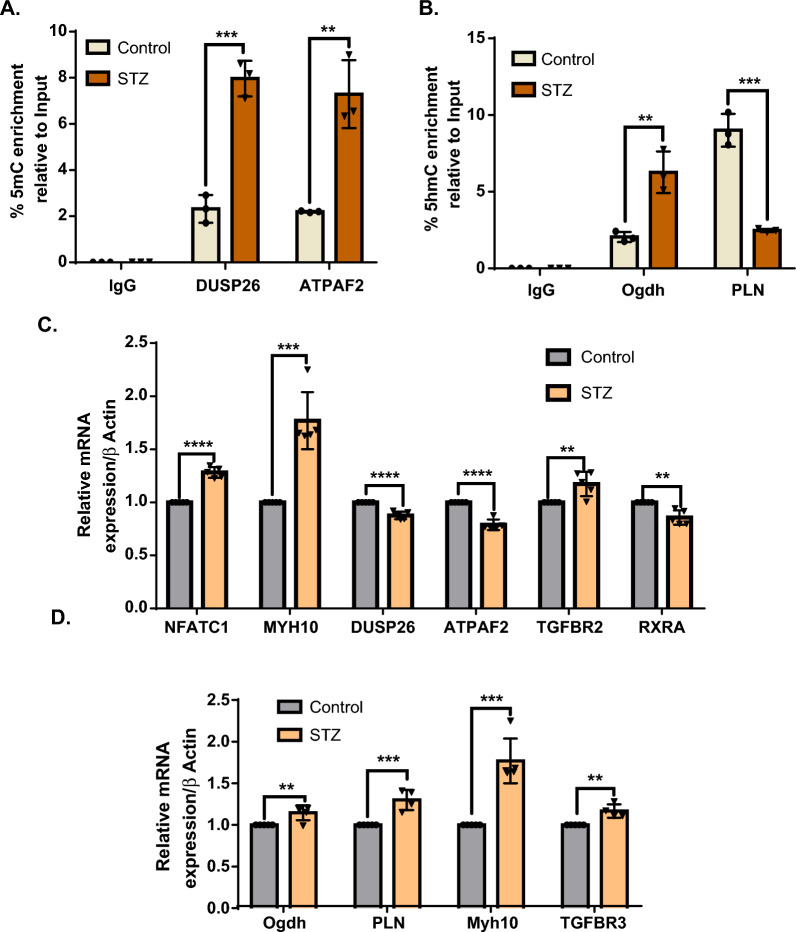
Validation of MEDIP/hMEDIP-seq data and functional relevance of 5mC/5hmC to gene expression. **A** Gene-specific enrichment of 5mC at DUDP26 and ATPAF2 in control and diabetic rats. **B** Gene-specific enrichment of 5hmC in the OGDH and PLN in control and diabetic rats. (n = 3). **C**, **D** qPCR analysis of relative mRNA levels in LV tissue of control and diabetic rat **C** For genes enriched in MEDIP-seq analysis, **D** For genes enriched in hMEDIP-seq analysis (n = 5) primers sequences are given in Additional file 1: table S5. For methylation and hydroxymethylation analysis, the relative enrichment of 5mC and 5hmC, respectively, was normalized to 10% input, and mRNA expression was normalized to the β-actin gene. The results are expressed as the mean ± SD of replicate values. Statistical significance was determined by Student’s unpaired t-test where *p < 0.05, **p < 0.001, ***p < 0.0002, ****p < 0.0001 with respect to control

To verify the reliability of our (h)MEDIP-seq data, we performed (h)MEDIP-qPCR for the genes showing significant differential methylation and hydroxymethylation. DUSP26 and ATPAF2 promoters showed increased methylation in MEDIP-qPCR analysis. The hMEDIP-qPCR results showed hyperhydroxymethylation in the promoter regions of OGDH and hypohydroxymethylation in the promoter regions of PLN in the hearts of diabetic rats (Fig. 3A and B). All these results were consistent with the (h)MEDIP-seq data.

To study the functional implications of 5mC and 5hmC accumulation in different genic regions, we performed gene expression analysis by real-time PCR for the selected genes based on their significant enrichment and pathological relevance to DCM. We found that the accumulation of 5mC modifications in the promoter region of the DUSP26 and ATPAF2 genes had a negative effect on their expression. Additionally, TGFBR2 and Myh10 showed hypermethylation in distal intronic regions with increased gene expression. On the other hand, expression of the NFATC1 gene was increased with decreased methylation levels in its first intron. Moreover, hypermethylation of the RXRA gene at multiple sites showed diminished expression in the diabetic heart (Fig. 3C and Additional file 1: Fig. S2).

The presence of 5hmC marks at the promoter or gene body regions of OGDH, Myh10, and TGFBR3 showed a positive correlation with gene expression (Fig. 3D). However, for the PLN gene, the level of hydroxymethylation and gene expression showed an inverse correlation, i.e., removal of 5hmC marks from the TSS site of the PLN gene in the diabetic heart increased its expression. The dual role of the 5hmC marks located within promoter regions in gene regulation was also reported previously [25, 26]. Thus, the above results indicate that the presence of DNA methylation and hydroxymethylation in the gene body regions alters its expression in pathological conditions such as DCM.

### AKG treatment restored cardiac function and reversed the pathophysiological phenotype in diabetic animals

The physiological, serum, ECG and hemodynamic parameters were analyzed in control and diabetic rats with or without AKG treatment (Additional file 1: Table S4). There was a significant increase in blood glucose, serum triglycerides, and total cholesterol levels, with a concomitant decrease in serum insulin levels in diabetic animals. Increases in blood glucose and triglyceride levels were prevented significantly upon AKG treatment in the diabetic group. Additionally, a significant increase in the heart weight to body weight ratio and cardiac damage marker CPK was observed in diabetic animals, which were improved upon supplementation with AKG.

Electrocardiographic analysis showed decreased heart rate with prolonged RR interval in diabetic animals. Both of these parameters were improved in AKG-treated diabetic animals. Changes in hemodynamic parameters such as arterial pressure, systolic and diastolic duration, maximum and minimum rate of change of pressure, and Tau indicated that cardiac function is compromised due to diabetes. AKG treatment restored the diastolic and systolic duration along with Tau in diabetic animals, which indicates the restoration of left ventricular function.

**Fig. 4:**
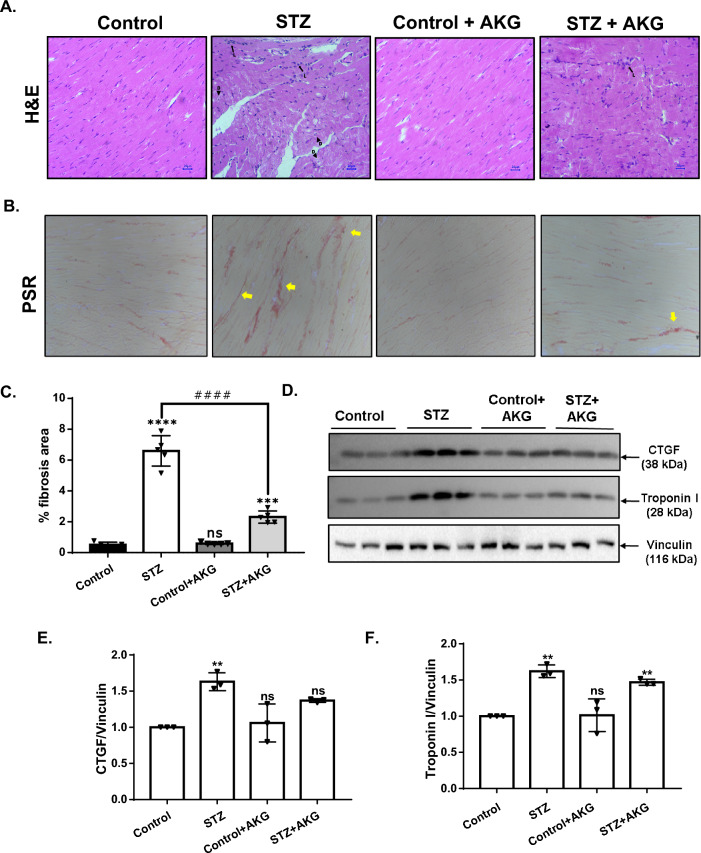
Effect of AKG supplementation on diabetes-induced myocardial damage and cardiac fibrosis. **A** Representative H&E-stained cardiac sections; arrows indicate lymphocyte infiltration, and arrowheads indicate degeneration of myocardial fibers. **B** Representative Sirius red-stained sections of cardiac tissue and deposition of collagen appear red as indicated by arrows. **C** Quantification of % fibrotic area in LV tissue(n = 5). **D** Western blot image depicting the protein levels of CTGF and troponin I. The bar graph below represents the relative levels of **E** CTGF and **F** troponin I in the heart tissue lysates of control and diabetic rats with or without AKG treatment (n = 3). Protein levels are normalized to vinculin. All values are given as the mean ± SD. Statistical significance was determined by one-way ANOVA with Tukey’s multiple comparisons post-test, where ***p < 0.0002, ****p < 0.0001 vs control group, ####p < 0.0001 vs STZ group

Cardiac fibrosis and histological examination were also performed in all the experimental groups. Histopathological analysis by H&E staining indicated no abnormality in the control animals with or without AKG treatment. While, there was moderate degeneration of cardiomyocytes with multifocal distribution of immune cells into the diabetic rat heart, AKG treated diabetic animals showed minimal degeneration of cardiomyocytes with focal distribution of immune cells (Fig. 4A) additional micrographs are shown in Additional file 1: Fig. S3. Increased interstitial fibrosis in diabetic heart tissue was evident by collagen deposition, as shown by picrosirius red staining (Fig. 4B and C). Additionally, fibrosis marker protein CTGF and cardiac damage marker protein troponin I levels were higher in diabetic rats than in controls (Fig. 4D, E, F). Along with the restoring cardiac function, AKG treatment also decreased myocardial fibrosis and cardiomyocyte degeneration in diabetic animals.

### Diabetes altered the expression of the enzymes participating in the DNA methylation and demethylation cycle

**Fig. 5:**
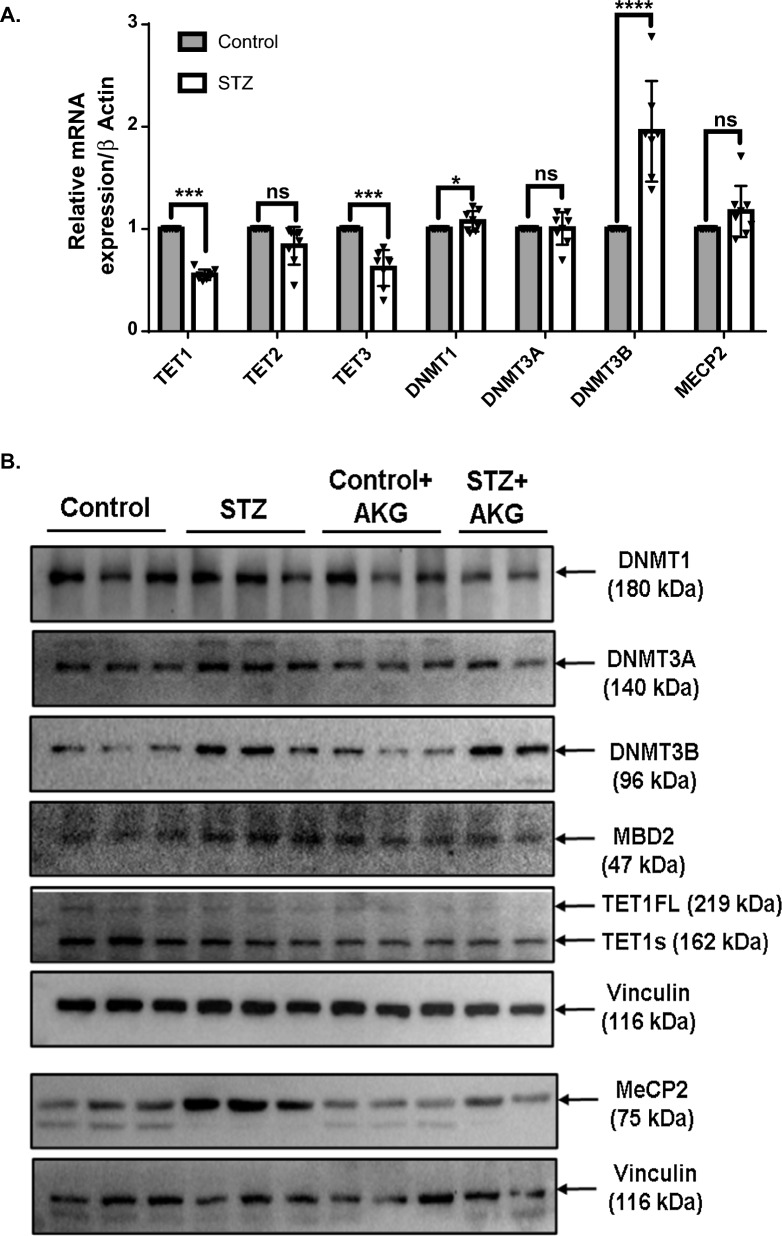
Expression of enzymes involved in the DNA methylation/demethylation cycle in diabetic LV tissue. **A** Relative mRNA levels of TET1, TET2, TET3, DNMT1, DNMT3A, DNMT3B, and MeCP2 in the LV of control and diabetic rats by qPCR are shown (n = 8). Data were normalized to β-actin. **B** Western blot analysis of 3 independent LV tissue lysates from control and diabetic rats with or without AKG treatment. Blots were probed with DNMT1, DNMT3A, DNMT3B, MBD2, TET1, and MeCP2 antibodies. Vinculin was used as a loading control. Densitometric analysis of western blot data is shown in the Additional file 1: Fig. S4. The results are represented as mean ± SD, Statistical significance was determined by Student’s unpaired t-test for real-time PCR, where *p < 0.05, ***p < 0.0002, ****p < 0.0001 with respect to control

Since the establishment and maintenance of the DNA (hydroxy)methylation patterns are depend on the function and coordination of different protein factors, we checked their transcript and protein levels. Real-time PCR analysis showed that there was a significant alteration in the mRNA levels of the enzymes taking part in the DNA methylation/demethylation cycle (Fig. 5A). We found a significant increase in the mRNA levels of DNMT1 and DNMT3B, while TET1 and TET3 transcript levels were significantly reduced.

However, we did not find any significant change in the mRNA levels of DNMT3A, MeCP2, and TET2 between diabetic and control rats. On the other hand, western blot analysis showed that DNMT3B, MeCP2, and MBD2 protein levels were significantly increased with no changes in DNMT1, DNMT3A, or TET1 protein expression (Fig. 5B and Additional file 1: Fig. S4). AKG supplementation in diabetic animals lowered the expression of MeCP2; however, it did not show a significant change in the expression of the other enzymes taking part in the DNA methylation and demethylation cycle. Thus, these results indicate that diabetes has the potential to alter the expression of enzymes that are part of the DNA methylation machinery; therefore, it can also alter the methylation levels in cardiac cells.

### Decreased global accumulation of 5mC/5hmC with increased TET1 DNA binding activity following AKG treatment

**Fig. 6:**
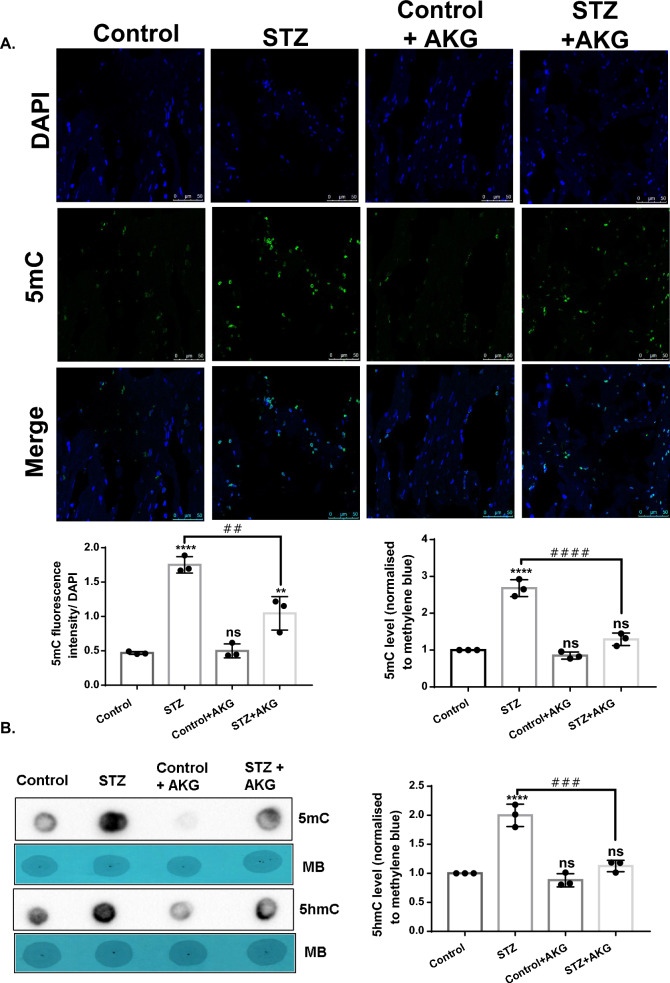
Global loss of 5mC and 5hmC in AKG-treated diabetic rats. **A** Representative images of immunohistochemical staining with 5mC antibody in cryosections of control and diabetic rats with or without AKG treatment and below is shown 5mC fluorescence intensity (Green) normalized to DAPI staining (Blue). **B** 5mC- and 5hmC-specific dot blot analysis of gDNA isolated from control and diabetic rats with or without AKG treatment. Equal loading of gDNA was assessed by methylene blue staining. Densitometry analysis of dot blots is shown with dot intensities normalized to methylene blue staining. The results are expressed as the mean ± SD in three different biological replicates. Statistical significance was determined by one-way ANOVA with Tukey’s multiple comparisons post-test, where *p < 0.05, **p < 0.001, ****p < 0.0001 vs control group, ##p < 0.001 ###p < 0.0002, ####p < 0.0001 vs STZ

Immunohistology staining and dot blot analysis revealed that AKG reversed the global accumulation of 5mC and 5hmC in diabetic LV tissue (Fig. 6A and B).

**Fig. 7:**
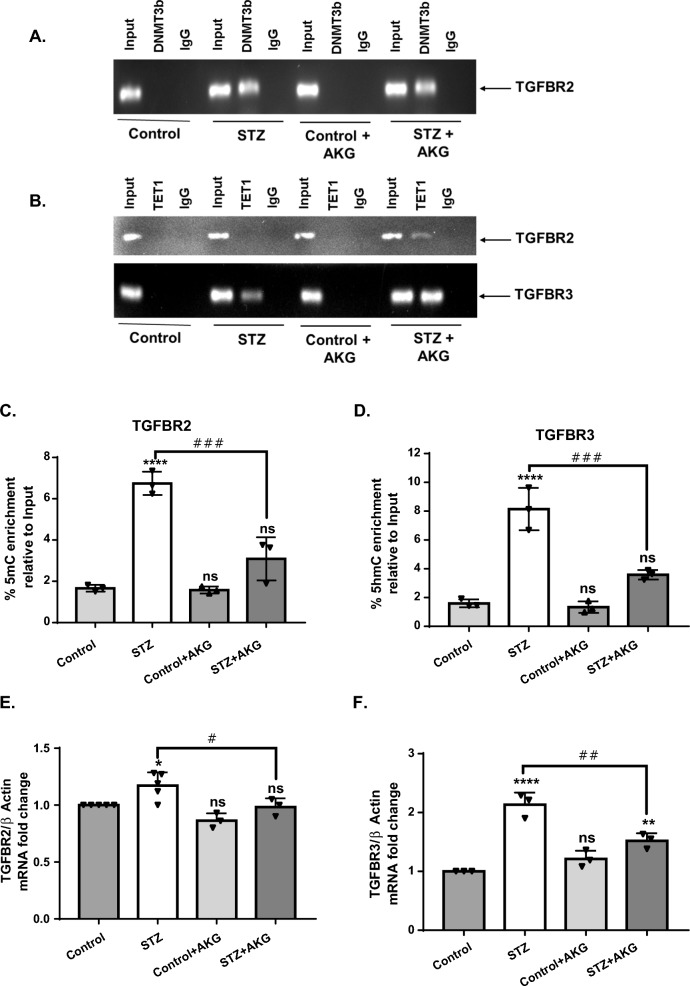
AKG increases TET1 binding in TGFBR2/TGFBR3 genes with decreased methylation/hydroxymethylation and expression in diabetic rats. **A**, **B** The binding activity (enrichment) of **A** DNMT3B to TGFBR2 and **B** TET1 to TGFBR2 and TGFBR3 intronic regions in control and diabetic rats with or without AKG treatment. Gene-specific enrichment of **C** 5mC in TGFBR2 and **D** 5hmC in TGFBR3 intronic regions with or without AKG treatment. **E**, **F** Relative mRNA levels of **E** TGFBR2 and **F** TGFBR3 in the LV of control and diabetic rats with or without AKG treatment by qPCR analysis. For methylation and hydroxymethylation analysis, the relative enrichment of 5mC and 5hmC, respectively, was normalized to 10% input, and gene expression was normalized to β-actin. The results are expressed as the mean ± SD in three different animals. Statistical significance was determined by one-way ANOVA with Tukey’s multiple comparisons post-test, where *p < 0.05, **p < 0.001, ****p < 0.0001 vs control group, #p < 0.05, ##p < 0.001 ###p < 0.0002 vs STZ

Moreover, (h) MEDIP-seq analysis revealed increased deposition of 5mC and 5hmC in the intronic regions of TGFBR2 and TGFBR3, respectively, in heart tissues of diabetic animals (Additional file 1: Fig. S2). Interestingly, ChIP-PCR analysis showed increased binding activity of DNMT3B to the intronic region of TGFBR2 in the diabetic heart, which was not altered in the presence of AKG (Fig. 7A). However, AKG treatment not only increased TET1 binding to TGFBR3 but also TGFBR2 in diabetic rats (Fig. 7B). These findings indicate that diabetes alters the binding activity of cytosine-modifying enzymes to genomic DNA and that AKG helps cardiac cells correct them.

Additionally, gene-specific (h)MEDIP-qPCR analysis showed that AKG treatment in diabetic animals lowered the enrichment of methylation and hydroxymethylation in the TGFBR2 and TGFBR3 intronic regions, respectively (Fig. 7C and D). This was accompanied by a decrease in the expression of TGFBR2 and TGFBR3 in these animals (Fig. 7E and F). These results strongly suggest that in addition to reversing the aberrant global 5mC/5hmC marks, AKG treatment also normalized the gene-specific (hydroxy)methylation patterns and subsequent gene expression in diabetic animals.

### DNMT3B knockdown exerts similar effects as AKG treatment in H9c2 cells exposed to hyperglycemia

**Fig. 8:**
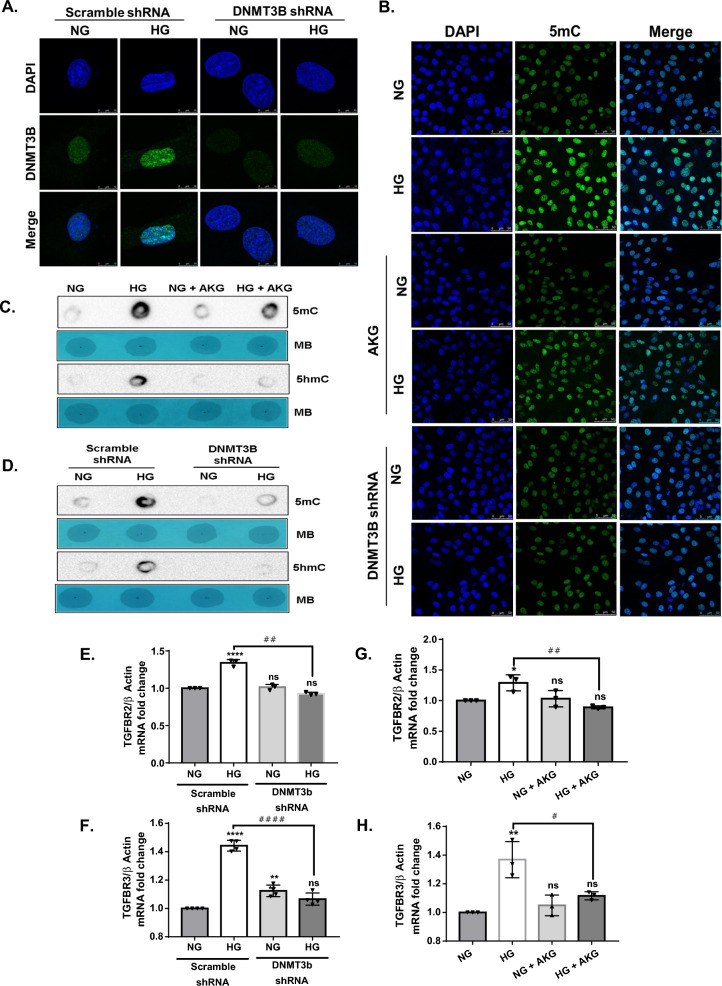
DNMT3B knockdown and AKG treatment inhibit DNA methylation/hydroxymethylation in H9c2 cells under hyperglycemic conditions. **A** Representative images of immunofluorescence staining with DNMT3B antibody in knockdown cells treated with normal glucose (NG) concentration (5.5 mM) and high glucose (HG) concentration (33 mM). **B** Representative images of immunofluorescence staining with 5mC antibody (Green) and nuclei stained with DAPI (Blue) in both DNMT3B knockdown and AKG-supplemented H9c2 cells in the presence of NG or HG. **C**, **D** 5mC- and 5hmC-specific dot blot analysis of gDNA isolated from NG- and HG-treated H9c2 cells in the presence of **C** AKG treatment and **D** DNMT3B knockdown. Equal loading of gDNA was assessed by methylene blue (MB) staining. Quantitative analysis of dot blot densitometry and 5mC immunofluorescence intensities are shown in the Additional file 1: Fig. S5. Relative mRNA levels of **E** TGFBR2 and **F** TGFBR3 in NG- and HG-treated H9c2 cells with or without AKG by qPCR analysis. Relative mRNA levels of **G** TGFBR2 and **H** TGFBR3 in NG- and HG-treated DNMT3B knockdown H9c2 cells by qPCR analysis. mRNA expression was normalized to β-actin, and the results are represented as the mean ± SD for three independent experiments. Statistical significance was determined by one-way ANOVA with Tukey’s multiple comparisons post-test, where *p < 0.05, **p < 0.001, ****p < 0.0001 vs NG (5.5 mM) treatment, #p < 0.05, ##p < 0.001 ####p < 0.0001 vs HG (33 mM) treatment

Since western blot and qPCR data showed an increase in the gene and protein expression of DNMT3B in the diabetic heart and in high glucose-treated H9c2 cells (Fig. 8A), we speculated that DNMT3B is involved in glucose-induced DNA hypermethylation. Hence, we next sought to confirm the involvement of DNMT3B in these altered DNA methylation patterns. We stably knocked down the expression of DNMT3B in H9c2 cells. Dot blot and immunofluorescence analyses showed that DNMT3B knockdown efficiently abolished hyperglycemia-induced global accumulation of 5mC/5hmC. Interestingly, we found a similar reversal of 5mC and 5hmC accumulation in high glucose-treated H9c2 cells supplemented with AKG (Fig. 8B, C, D). Furthermore, the transcript levels of TGFBR2 and TGFBR3 were increased in high glucose-treated cells. However, DNMT3B knockdown (Fig. 8E and F) and AKG treatment (Fig. 8G and H) in high glucose-treated H9c2 cells did not increase the expression of these two genes. The role of DNMT3B in glucose-induced hyper(hydroxy)methylation is evident from these results. Additionally, the potential of AKG as an epigenetic modulator in hyperglycemia is recognized.

## Discussion

This study demonstrated that diabetes induces genome-wide accumulation of 5mC and 5hmC in left ventricular tissue. Although a few studies have already shown methylation maps in diabetic cardiac tissue, this is the first study, to our knowledge, giving a comprehensive understanding of diabetes-induced genome-wide alterations in hydroxymethylation patterns during the progression of DCM. Most of these cytosine modifications are enriched in the genes involved in pathophysiological signaling pathways promoting fibrosis, cardiomyocyte hypertrophy, and metabolic perturbations in the diabetic heart. Furthermore, we have shown that AKG supplementation in diabetic animals can improve cardiac function through epigenetic modulation. Our study also demonstrated that the de novo methyltransferase DNMT3B could mediate these epigenetic alterations in cardiac tissue of diabetic animals, as confirmed by in vitro knockdown of DNMT3B in H9c2 cells, which prevents hyperglycemia-induced accumulation of 5mC and 5hmC.

Our (h)MEDIP-seq analysis revealed that increased glucose levels promote overall hypermethylation and hyperhydroxymethylation in the cardiac tissue of diabetic animals. Spallota et al*.* also reported a similar observation, who showed that 5mC and its oxidative intermediate product 5hmC accumulate in cardiac mesenchymal cells isolated from T2D patients, cardiac tissue of STZ-induced diabetes and HFD-fed mice [19]. The current study also demonstrated that the genomic distribution of the 5mC and 5hmC peaks was significantly enriched in gene body regions, particularly in intronic regions (Fig. 1). The presence of 5mC or 5hmC marks at gene body regions was reported to play an important role in alternative RNA splicing [27, 28]. Alternative RNA splicing defects and the possible implications of 5mC and 5hmC deposition in the intronic regions of the genes for controlling these defects have already been reported in other cardiac diseases [29–31]. Thus, the accumulation of the cytosine modification in the gene body regions can employ a novel mechanism in elevating the pathogenic response under diabetic conditions. Hence, we performed KEGG pathway enrichment analysis for the genes showing the presence of DMRs and DhMRs in the gene body regions. The most significantly enriched pathways for hyper-5mC were calcium signaling, Rap1 signaling, apelin signaling, and phosphatidylinositol signaling, while hypo-5mC was enriched for calcium signaling. Hyper-5hmC was found in pathways related to metabolic perturbations, cellular senescence, and calcium signaling. While the calcium signaling pathway was enriched for 5mC and 5hmC D(h)MRs, the regions enriched in both analyses differed. This observation indicates that the occurrence of these two types of cytosine modifications is independent of each other in current experimental conditions.

Furthermore, the functional implications of these epigenomic alterations on gene expression in DCM were elucidated by real-time PCR analysis. Pathological perturbations in the molecular pathways during the progression of cardiac disorders, including DCM, were also described previously [32–38]. Thus, our KEGG pathway analysis of 5mC and 5hmC enrichment in the gene body regions and their effect on the expression of the associated genes explains the epigenomic basis of aberrant activation of signaling pathways.

Depletion of AKG in diabetes and obesity was reported earlier [23, 24, 39]. Considering the current findings of our study and the importance of AKG in active DNA demethylation, we hypothesize that supplementation of AKG in diabetic rats can impede the pathological outcomes of hyperglycemia on the structure and function of the heart tissue. Our ChIP-PCR data showed the increased binding activity of TET1 to the genomic regions of TGFBR2 and TGFBR3 in the heart tissue of diabetic rats supplemented with AKG. MEDIP-qPCR data also demonstrated the removal of 5mC marks from TGFBR2 and 5hmC marks from TGFBR3 with a concomitant decrease in gene expression. The role of TGFBR2 and TGFBR3 in cardiac fibrosis was previously reported [40, 41]. These observations suggest that AKG might have a protective role in the cardiac remodeling of a diabetic heart through epigenetic modulation and suppression of profibrotic genes. AKG treatment also reduced the global accumulation of 5mC and 5hmC, the intermediate product of active DNA demethylation. This finding can be explained by the fact that along with acting as a cofactor for TET enzymes and promoting their binding activity to methylated DNA, AKG also functions as an allosteric activator of TDG and enhances the demethylation process by increasing the association of the TET-TDG complex for the complete removal of modified cytosine [23]. One of the interesting findings of the current study is that AKG treatment significantly lowered the blood glucose levels in diabetic rats. Recent findings by Yuan et al. have also shown the hypoglycemic effect of AKG by inhibiting hepatic gluconeogenesis [42]. These observations suggest that along with reversing aberrant methylation marks from key genes involved in pathological signaling pathways, AKG may also help the diabetic heart overcome molecular insults imposed by hyperglycemia by maintaining glucose homeostasis.

Furthermore, in the diabetic heart, increased DNA methylation is also accompanied by an increase in the expression of de novo methyltransferase DNMT3B along with an increase in the expression of MeCP2, and MBD2, which can explain the accumulation of 5mC in diabetic conditions. DNMT3B knockdown in H9c2 cells treated with high glucose confirmed the role of this methyltransferase enzyme in hyperglycemia-induced increase in the DNA methylation levels. Similarly, Vigorelli et al. also reported the involvement of DNMTb-induced CXCR4 gene promoter methylation and gene repression in HG-treated cord blood-derived CD34^+^ stem cells and the bone marrow-derived CD34^+^ stem cells isolated from patients having coronary artery disease along with diabetes mellitus [43]. Additionally, Wei and Loeken showed that in the presence of hyperglycemia, DNMT3B activity was increased due to an increase in oxidative stress which is responsible for methylation of CpG island and gene silencing of Pax3 gene during neurulation of embryonic stem cells and in embryos isolated from the hyperglycemic pregnant mice [44]. AKG treatment in hyperglycemia-exposed H9c2 cells also showed a similar reversal of global 5mC and 5hmC deposition, possibly by improving TDG function and its association with TET1 to complete the DNA demethylation cycle. Thus, our data suggest that the role of DNMT3B is indispensable in high glucose-induced cytosine modifications. Additionally, the potential of AKG to modulate genome-wide as well as gene-specific methylation marks is evident in the current study.

The present study could serve as a basis for understanding the genome-wide differences in 5mC and 5hmC patterns and their functional relevance in the disease phenotype of DCM at the molecular level. However, this study has some limitations. Our bulk tissue analysis limits the scope for determining cell type-specific changes in methylome and hydroxymethylome patterns. However, for this analysis, we used only the apical region of the heart tissue to maintain cellular homogeneity among all the experimental samples. Furthermore, AKG is a pleiotropic molecule that can act as a cofactor for chromatin-modifying enzymes, including TETs and histone demethylases. Future studies exploring the use of AKG for therapeutic intervention in the treatment of DCM should thus employ an integrated approach with the global transcriptome, histone methylome, and DNA methylome to obtain an in-depth understanding of AKG treatment with phenotypic rescue in the diabetic heart.

## Conclusion

In conclusion, this study indicates that diabetes encrypts aberrant 5mC and 5hmC marks on the genomic DNA of the diabetic heart, which is accompanied by increased expression of DNMT3B, MeCP2, and MBD2. AKG supplementation helps to ameliorate these aberrant methylation patterns, increases the binding activity of TET at gene-specific sites, and enhances the process of DNA demethylation. This, in turn, may result in decreased cardiac fibrosis and restoration of cardiac function by reversing the gene expression changes and pathological signaling imposed by diabetes on the heart tissue. Although conventional therapies restore blood glucose levels, they fail to improve the progression of DCM. The current study, however, suggests the potential use of epigenetic drug therapies such as AKG for the effective management of chronic complications of diabetes and for delaying disease progression.

## Methods

### Animal model

Six- to eight-week-old male Wistar rats were used for this study (n = 8). All procedures on animals were performed according to the institutional animal ethical committee guidelines (IAEC) of NCCS. Streptozotocin (STZ, Sigma-Aldrich) dissolved in citrate buffer (pH 4.5) was given to the animals at a dose of 55 mg/kg body weight to induce diabetes. Control animals received an equivalent volume of citrate buffer. After confirmation of diabetes, control and diabetic animals were randomized into four groups with or without AKG treatment. The treatment groups received a daily oral dose of 250 mg/kg body weight AKG (Sigma-Aldrich) until the end of the experiment. The total duration of the experiment was three months.

### Echocardiography and hemodynamic parameters

Rats were anesthetized using urethane (1 g/kg BW, i.p.) at the end of the experiment. ECG was recorded using ECG surface electrodes (AD Instruments, Colorado Springs, CO). A microtip pressure transducer catheter (SPR-320, Millar Instruments, Houston, TX, United States) was advanced into the left ventricles to record the hemodynamic parameters. The catheter was connected to an 8-channel Powerlab instrument via a bridge amplifier (AD Instrument). Data were recorded and analyzed by Lab Chart 7 software (AD Instrument). Left ventricular apical tissue was either snap frozen, embedded in OCT compound for IHC, or immersed in buffered formalin. Blood was collected from the abdominal aorta, and serum was isolated and stored at − 80 °C until further analysis.

### Serum analysis

Insulin levels were measured by an ELISA kit (Mercodia AB, Sweden). Total cholesterol, triglycerides, and cardiac tissue damage markers creatine phosphokinase (CPK) and creatine kinase-myocardial band (CK-MB) were analyzed.

### Histological staining

Formalin-fixed heart tissues were embedded in paraffin wax. Sections of 3–4 µm thickness were taken with a rotary microtome (Leica, Germany) and stained with hematoxylin & eosin (H & E) reagent. Prepared slides were examined under a microscope (Olympus, Japan), and images were taken at 40X magnification to note histopathological lesions. The severity of the lesions was determined by grading system; as 0 = within normal limits, 1 = Minimal, 2 = Mild/Slight, 3 = Moderate, 4 = Severe, and distribution of lymphocytic infiltration was recorded as focal, multifocal, and diffuse at ventricular myocytes as described previously [45].

### Picrosirius red staining

Interstitial tissue collagen deposition and fibrosis were analyzed by picrosirius red (PSR) staining. Sections were deparaffinized and serially rehydrated and stained with 0.1% PSR stain. Then, sections were mounted with DPX mounting medium and observed under a light microscope (Nikon, Tokyo, Japan) and images were taken at 40 × magnification to note collagen deposition.

### Cell culture

Embryonic cardiomyoblast H9c2 cells were maintained in Dulbecco’s Modified Eagle Medium (DMEM) supplemented with 10% serum. Upon reaching 70% confluency, cells were seeded into 100 mM dishes provided with DMEM containing 1% serum. These cells were treated with normal glucose (NG, 5.5 mM glucose) or high glucose (HG, 33 mM glucose) for 72 h with or without AKG (3.5 mM) treatment.

### DNMT3B knockdown

A short hairpin RNA (shRNA) sequence targeting DNMT3B was cloned into the pLKO.1—TRC vector. Lentiviral particles were generated by transfecting HEK293FT cells with the DNMT3B shRNA construct along with packaging vectors (pMD2. G and psPAX2). A stable H9c2 cell line expressing shRNA was obtained by lentiviral infection followed by puromycin (1.5 µg) selection. The efficiency of DNMT3B knockdown was confirmed by western blot and qPCR analysis. Scrambled shRNA was used as a negative control. Oligonucleotide sequences of shRNA targeting DNMT3B are given in Additional file 1: Table S5.

### Immunohistochemical (IHC) and immunofluorescence (IF) studies for 5mC

Frozen heart sections and H9c2 cells were stained with an anti-5mC antibody (28692S, Cell Signaling Technology, USA) according to the manufacturer’s protocol for IHC and IF. In brief, sections and cells were treated with 70% ethanol for 10 min followed by treatment with 2 N HCl for 1 h. After blocking nonspecific antibody binding using 3% bovine serum albumin, specimens were incubated overnight at 4 °C with primary antibody (1:60 dilution). Subsequently, specimens were stained with anti-rabbit Alexa Fluor^®^ 488-conjugated secondary antibody (Invitrogen, USA). Nuclei were stained with 4′-6- diamidino-2-phenylindole (DAPI). Sections and cells were then mounted and imaged by a confocal laser scanning microscope (CFLSM) (Leica SP5II). Primary antibody was omitted for negative control experiments. For DNMT3B expression, the IF protocol was carried out as described previously [46]. Images were analyzed using ImageJ software (National Institutes of Health).

### Dot Blot

Genomic DNA from left ventricular apical tissue and H9c2 cells was isolated using a DNeasyTissue kit (Qiagen). Dot blot analysis was performed to determine the levels of 5mC/5hmC in gDNA as described by Ito S., et al. with some modifications [47]. In brief, 1 µg gDNA was sonicated and subjected to alkali denaturation at 95 °C for 10 min. Denatured DNA was spotted manually onto the Hybond-N + nylon membrane (Amersham Biosciences). Then, DNA was crosslinked to the membrane by UV irradiation at 1200 mJ/cm^2^ dosage followed by blocking with 5% nonfat dry milk and probing with 5mC (rabbit)/5hmC (mouse) antibody (Cell Signaling Technology, USA) overnight at 4 °C, followed by respective HRP-conjugated secondary antibodies and detection with a chemiluminescence kit. To ensure equal spotting of total DNA on the membrane, the same blot was stained with 0.05% methylene blue (MB) in 0.3 M sodium acetate (pH 5.2). Densitometric quantification of the nucleic acid dot blot intensities was performed with ImageJ software (National Institutes of Health), and the values were normalized to methylene blue staining.

### MeDIP/hMeDIP sequencing

Purified genomic DNA from tissue samples with two biological replicates from control and STZ groups was subjected to (h) MeDIP-seq library preparation. In brief, 5 μg of genomic DNA was fragmented by sonication to a mean size of approximately 200–400 bp, followed by end repair, A-base tailing, and adapter ligation. After adapter ligation, 10% of the DNA was secured, which served as input DNA. The remaining DNA was immunoprecipitated by using a MagMeDIP kit for 5mC and an hMEDIP kit for 5hmC (Diagenode). This captured DNA and input DNA were then purified with Zymo DNA Clean & Concentrator^™^-5 (Zymo Research) and subsequently amplified with 12 cycles using KAPA HiFi HotStart ReadyMix (Kapa Biosystems, Wilmington, MA, USA) and Illumina Multiplexing PCR Primers. An Agilent 2100 Bioanalyzer then analyzed the constructed libraries and finally sequenced on the Illumina platform (Illumina X Ten, paired-end, 150 bp).

### Data processing

The quality of raw data from MEDIP, hMEDIP, and input samples was evaluated with FASTQC, the adapter sequence was trimmed with the help of Trimmomatic software (v0.36). Clean reads were mapped on rat genome Rn6 (Rnor_6.0) using bowtie2 (v2.4.4) followed by peak calling by MACS2 (v2.2.7.1) compared to input. Differentially methylated regions (DMRs) and differentially hydroxymethylated regions (DhMRs) between control and diabetic animals were detected using PePr (v1.1.24) software with default parameters. PePr uses a window-based approach and models read counts across intragroup and intergroup replicates with a negative binomial distribution [48]. Differential peaks were annotated using ChipSeeker (v1.8.6). The promoter region is identified as a − 1 kb region from the transcription start site (TSS). Kyoto Encyclopedia of Genes and Genomes (KEGG) and Gene Ontology (GO) enrichment analyses were performed for the DMRs/DhMRs using PathfindR (v1.6.2) and ShinyGO (v0.76.3) software packages, and peaks were visualized using Integrative Genomics Viewer (IGV) (v2.15.2) software.

### MEDIP/hMEDIP-qPCR

Purified gDNA from ventricular tissue (1 µg for MEDIP and 2 µg for hMEDIP) was sonicated to obtain a fragment size of 300–500 bp by using Bioruptor pico (Diagenode). MEDIP was performed as described previously [49]. Methylated DNA was immunoprecipitated by using an anti-5mC antibody (286928, CST). Immunoprecipitation of 5hmC was performed with an hMEDIP kit (Diagenode) according to the manufacturer's protocol. After immunoenrichment, DNA was subjected to proteinase K digestion, resuspended in TE buffer, and used for qPCR analysis. The primers used for qPCR analysis are given in Additional file 1: Table S5. The following formula calculated percent enrichment relative to 10% input,

% 5mC/5hmC enrichment relative to input = 2^[(Ct(10% input)−3.32)−Ct(IP)] × 100%^

Where 3.32 is the compensatory factor for 10% input taken from the sample.

### Real-time PCR

Ventricular tissue or H9c2 cells were used to analyze gene expression by real-time PCR (qPCR). Total RNA was isolated using TRIzol^®^ reagent (Invitrogen, Carlsbad, CA, USA), and 1 µg of RNA was reverse transcribed using a Verso cDNA synthesis kit (Thermo Scientific, Waltham, MA, USA) according to the manufacturer’s protocol. qPCR was performed with the primers given in Additional file 1: Table S5 by using Power Up™ SYBR™ Green Master Mix (Applied Biosystems, Austin, TX, USA) on QuantStudio 3 (Applied Biosystems, Foster City, CA, USA). Gene expression was determined by normalization to β-actin by employing the ΔΔCt method.

### Western blotting

Frozen tissue samples and H9c2 cells were lysed in RIPA buffer and processed as described previously [46] for western blot analysis using the following primary antibodies: DNMT1 (ab13537, Abcam), DNMT3A (3598, Cell Signaling Technologies), DNMT3B (ab2851, Abcam), MeCP2 (3456, CST), MBD2 (sc-514062, Santa Cruz Technologies), TET1 (ab157004, Abcam), CTGF (sc-14939, Santa Cruz Technologies), Troponin I (4002, Cell Signaling Technologies), and Vinculin (V9131, Sigma). Immune complexes were detected by SuperSignal West Pico Plus substrate (Thermo Fisher, Carlsbad, CA, USA) on a Chemidoc XRS + system (Bio-Rad). Densitometric quantification of the band intensities was performed with ImageJ software (National Institutes of Health), and the values were normalized to those of Vinculin.

### Chromatin immunoprecipitation (ChIP)

To determine the binding activity of DNMT3B and TET1 to the (hydroxy)methylated regions, we performed ChIP-PCR as per the protocol described by Perna and Alberi [50] with some modifications. In brief, 35 mg of frozen tissue samples were minced and crosslinked with 1% formaldehyde solution for 15 min. The cross-linking reaction was stopped by adding 0.125 M glycine solution. Then, samples were ground in lysis buffer with protease inhibitor with the help of a Dounce homogenizer. Chromatin was sonicated to obtain a fragment size of 200–500 bp. One percent of the input DNA was stored at 4 °C. Protein concentration was determined by the Bradford method, and 30 µg of protein was used for immunoprecipitation for each sample. Immunoprecipitation of chromatin fragments was carried out using ChIP-grade antibodies against DNMT3B (ab2851, Abcam) or TET1 (ab157004, Abcam). Preimmune IgG was used as a negative control. Specific antibody–chromatin complexes were then precipitated by incubation with ChIP-grade protein-G-coated agarose beads (Invitrogen, USA) for 4 h at 4 °C. DNA samples were eluted, and supernatants were used for reverse cross-linking of the protein/DNA complexes to free DNA. The purified DNA was subjected to PCR amplification using TGFBR2 and TGFBR3 primer pairs (Additional file 1: Table S5) encompassing the sequence analysis. PCR products were visualized on 1.2% agarose gel.

### Statistical analysis

Data are expressed as the mean ± standard deviation (SD). Unpaired *t*-tests were used for comparisons between two groups, and one-way ANOVA with Tukey’s multiple comparisons post-test was used for multiple groups. GraphPad Prism software (GraphPad, San Diego, CA, USA) was used to analyze statistical significance. A value of *p* < 0.05 was considered statistically significant.

## Supplementary Information

**Additional file 1: Table S1.** KEGG pathway list for DMRs showing hypo-5mC in gene body regions of cardiac tissue of STZ vs control. **Table S2**. KEGG pathway list for DMRs showing hyper-5mC in gene body regions of cardiac tissue of STZ vs control. **Table S3**. KEGG pathway list for DhMRs showing hyper-5hmC in gene body regions of cardiac tissue of STZ vs control. **Table S4**. Physiological, cardiac, and serum parameters of experimental animals. **Table S5**. Primer and oligos sequences. **Fig. S1**. GO enrichment analysis. Significantly enriched GO terms in (A) MEDIP-seq and (B) hMEDIP-seq analysis. Red, green, and blue bars represent the biological process, cellular component, and molecular function respectively. The x-axis represents -log10(p-value), the y-axis represents GO terms and the number to the right of the column indicates the number of enriched genes. **Fig. S2**. Enrichment of DMRs and DhMRs within gene body regions visualized by IGV. Locations of the differentially methylated and hydroxymethylated peaks within gene body regions were visualized by IGV genome browser. Hypermethylated/ hyperhydroxymethylated peaks are denoted by black color and hypomethylated/ hypohydroxymethylated peaks are denoted by red color. Refseq gene is shown in the first track, hMEDIP peaks in the middle track, and MEDIP peaks are in the lower track. **Fig. S3**. H&E staining micrographs. H&E-stained cardiac sections from three different rat LV tissues from each group; arrows indicate lymphocyte infiltration, and arrowheads indicate degeneration of myocardial fibers. **Fig. S4**. Densitometry analysis of western blot data. Densitometry analysis of western blot data is shown in Fig. 5B for (A) DNMT1, (B) DNMT3A, (C) DNMT3B, (D) MeCP2, (E) MBD2, and (F) TET1. Band intensities are normalized to Vinculin and represented as mean ± SD for three different animals. Statistical significance was determined by one-way ANOVA with Tukey’s multiple comparisons post-test where, *p < 0.05, **p < 0.001, ****p < 0.0001vs control, and ###p < 0.0002 vs STZ by one-way ANOVA. **Fig. S5**. Quantitative analysis of global 5mC/5hmC levels in *in vitro* experiments. **(A, B) **Densitometry analysis of 5mC- and 5hmC-specific dot blot analysis of gDNA isolated from NG- and HG-treated H9c2 cells in the presence of AKG treatment (A) 5mC levels and (B) 5hmC levels. **(C, D) **Densitometry analysis of 5mC- and 5hmC-specific dot blot analysis of gDNA isolated from NG- and HG-treated DNMT3b kockdown-H9c2 cells (C) 5mC levels and (D) dot blot intensities are normalized to methylene blue staining. (E,F) Immunofluorescence intensities of 5mC antibody staining in presence of NG or HG in (E) AKG-supplemented and (F) DNMT3b knockdown H9c2 cells fluorescence intensities were normalised to the DAPI staining. Results are represented as mean ± SD for three individual experiments and statistical significance was determined by one-way ANOVA with Tukey’s multiple comparisons post-test where, **p < 0.001, ***p < 0.0002, ****p < 0.0001vs control, and ##p < 0.001, ###p < 0.0002, ####p < 0.0001 vs STZ.

## Data Availability

The datasets generated and/or analyzed during the current study are available in the additional file, and additional data can also be available from the corresponding author upon reasonable request.

## Abbreviations

DCM: Diabetic cardiomyopathy
STZ: Streptozotocin
LV: Left ventricle
MEDIP: Methylated DNA immunoprecipitation
hMEDIP: Hydroxymethylated DNA immunoprecipitation
(h) MEDIP: MEDIP and hMEDIP
DMR: Differentially methylated regions
DhMR: Differentially hydroxymethylated regions
D(h)MR: DMR and DhMR
(hydroxy)methylation: Methylation and hydroxymethylation
TSS: Transcription start site
chip-PCR: Chromatin immunoprecipitation-PCR
RT-qPCR: Reverse transcription-quantitative real-time polymerase chain reaction
IHC: Immunohistological chemistry
PSR: Picrosirius red
5mC: 5-Methylcytosine
5hmC: 5-Hydroxymethylcytosine
HG: High glucose
NG: Normal glucose
DMEM: Dulbecco’s modified eagle medium
MB: Methylene blue
AKG: Alpha-ketoglutarate
DNMT: DNA methyltransferase
TET: Tet Eleven Translocase
TDG: Thymine DNA glycosylase
MeCP2: Methyl CpG binding protein 2
MBD2: Methyl-CpG binding domain protein 2
CTGF: Connective tissue growth factor
TGFBR: Transforming growth factor, beta receptor
OGDH: Oxoglutarate dehydrogenase
PLN: Phospholamban
Myh10: Myosin heavy chain 10
ATPAF2: ATP synthase mitochondrial F1 complex assembly factor 2
Slc2a3: Solute carrier family 2 member 3
DUSP26: Dual specificity phosphatase 26
RXRA: Retinoid X receptor alpha
NFATC1: Nuclear factor of activated T cells 1
KEGG: Kyoto Encyclopedia of Genes and Genomes
GO: Gene ontology
