# Supplementary material for: Epigenetic modifier alpha-ketoglutarate modulates aberrant gene body methylation and hydroxymethylation marks in diabetic heart
Source: Epigenetics Chromatin. 2023 Apr 27;16:12. doi: 10.1186/s13072-023-00489-4 (PMC10134649; doi:10.1186/s13072-023-00489-4)

**Additional file**

**Epigenetic modifier alpha-ketoglutarate modulates aberrant gene body methylation and hydroxymethylation marks in diabetic heart**

Rohini Dhat^1^, Dattatray Mongad^2^, Sivarupa Raji^1^, Silpa Arkat^3^, Nitish R. Mahapatra^3^, Nishant Singhal^1^  and Sandhya L. Sitasawad^1^

1. National Centre for Cell Science, NCCS Complex, S.P. Pune University, Ganeshkhind, Pune 411007, Maharashtra, India.
2. NCMR-National Centre for Cell Science (NCCS), Pune, Maharashtra 411007, India.
3. Department of Biotechnology, Bhupat and Jyoti Mehta School of Biosciences, Indian Institute of Technology Madras, Chennai, 600036, India.

**Table of contents**

***Table S1 – KEGG pathway list for DMRs showing hypo-5mC in gene body regions………3***

***Table S2 – KEGG pathway list for DMRs showing hyper-5mC in gene body regions…...…3***

***Table S3 – KEGG pathway list for DhMRs showing hyper-5hmC in gene body regions…..4***

***Table S4. Physiological, cardiac, and serum parameters of experimental animals……...5-6***

***Table S5 - Primer and oligo sequences………………………………………………….…7-8***

***Fig. S1 - GO enrichment analysis…………………………………………………….…9-10***

***Fig. S2 - Enrichment of DMRs and DhMRs within gene body regions visualized by IGV.11***

***Fig. S3- H&E staining micrographs………………………………………………………..12***

***Fig. S4 - Densitometry analysis of western blot data…………………………………….....13***

***Fig. S5 -* Quantitative analysis of global 5mC/5hmC levels in *in vitro* experiments*...14-15***

| **Pathway** | **-log10(FDR)** | **Gene list** |
| --- | --- | --- |
| Calcium signaling pathway | 4.06 | Flt1, Pdgfa, Itpr2, Cacna1c, Plcz1, Ppp3ca, Slc25a4, Camk2d, Trdn, Gdnf, Erbb4, Gna14, Grm5, Grin2a, Cckar, Tacr2, Plcg2, Cacna1g, Atp2b3 |
| Axon guidance | 3.99 | Sema5b, Srgap1, Ppp3ca, Camk2d, Sema5a, Ntng2, Wnt5a, Sema4c, Bmpr1b, Rgs3, Robo2, Robo1, Lrrc4c, Ntng1, Efna5, Plcg2 |
| Dopaminergic synapse | 2.20 | Itpr2, Ppp1cb, Gria4, Cacna1c, Ppp3ca, Camk2d, Arntl, Ppp2r2b, Akt3, Grin2a, Ppp2r2c |
| Mucin type O-glycan biosynthesis | 2.00 | C1galt1, Galnt14, B4galt5, Galnt7, Galnt17 |
| Long-term potentiation | 2.00 | Itpr2, Ppp1cb, Cacna1c, Ppp3ca, Camk2d, Grm5, Grin2a |
| Phospholipase D signaling pathway | 2.00 | Pdgfa, Sos2, Syk, Grm5, Agpat4, Gab1, Pip5k1c, Akt3, Dgki, Agpat5, Plcg2 |
| Cellular senescence | 2.00 | Foxo3, Itpr2, Fbxw11, Ppp1cb, Rassf5, Foxm1, Ppp3ca, Slc25a4, Btrc, Nfatc1, Akt3, Atm |
| Cell adhesion molecules | 2.00 | Vsir, Alcam, Nrcam, Cldn10, Ntng2, Vtcn1, Pvr, Vcan, Lrrc4c, Ntng1, Cd226 |
| Proteoglycans in cancer | 2.00 | Itpr2, Ppp1cb, Sos2, Camk2d, Erbb4, Wnt5a, Gab1, Ezr, Stat3, Vav3, Akt3, Itgb3, Plcg2 |
| Aldosterone synthesis and secretion | 1.79 | Scarb1, Itpr2, Cacna1c, Cyp11a1, Kcnk3, Camk2d, Cacna1g, Atp2b3 |

**Table S1 KEGG pathway list for DMRs showing hypo-5mC in gene body regions of cardiac tissue of STZ vs control**

The top 10 most significant path entries for enrichment are depicted.

**Table S2 KEGG pathway list for DMRs showing hyper-5mC in gene body regions of cardiac tissue of STZ vs control**

| **Pathway** | **-log10(FDR)** | **Gene list** |
| --- | --- | --- |
| Calcium signaling pathway | 5.72 | Flt1, Mcoln1, Nos1, P2rx4, Adora2a, Mylk, Pdgfra, Stim2, Atp2b4, Egfr, Plcb1, Tacr1, Cacna1c, Mylk2, Slc8a1, Plcz1, Gnal, Sphk1, Ednrb, Gdnf, Cacna1d, Gnaq, Erbb4, Cysltr2, Fgfr1, Lhcgr, Agtr1a, Pdgfrb, Ptk2b, Plcd1, Plcb4, F2r, Plcg2, Cacna1g |
| Rap1 signaling pathway | 5.02 | Flt1, Adora2a, Pdgfra, Egfr, Map2k6, Plcb1, Lcp2, Vav2, Epha2, Lpar1, Fyb1, Mras, Gnaq, Igf1r, Fgfr1, Efna2, Pdgfrb, Pik3r2, Magi3, Vav3, Sipa1, Tiam1, Magi1, Skap1, Insr, Pard3, Rasgrp3, Plcb4, Efna5, F2r |
| Phosphatidylinositol signaling system | 3.95 | Mtm1, Plcb1, Mtmr3, Plcz1, Synj2, Dgkz, Pik3c3, Inpp4b, Impa2, Inpp5e, Pik3r2, Dgki, Dgkb, Plcd1, Plcb4, Pik3c2g, Plcg2 |
| Phospholipase D signaling pathway | 3.89 | Plpp2, Pdgfra, Egfr, Plcb1, Grm7, Sphk1, Grm2, Lpar1, Mras, Agpat4, Dgkz, Agtr1a, Pdgfrb, Pik3r2, Dgki, Ptk2b, Pld1, Insr, Dgkb, Plcb4, F2r, Plcg2 |
| Apelin signaling pathway | 3.61 | Nos1, Prkab1, Mylk, Plcb1, Slc9a1, Mylk2, Slc8a1, Smad3, Sphk1, Mras, Gnaq, Plin1, Gng8, Pik3c3, Agtr1a, Gng7, Hdac4, Lipe, Mef2d, Plcb4 |
| Yersinia infection | 3.19 | Limk1, Mapk10, Actr3, Map2k4, Map2k6, Actr2, Lcp2, Traf2, Vav2, Skap2, Fyb1, Gnaq, Cd4, Arhgef28, Pik3r2, Vav3, Nfkb1, Ptk2b |
| Gastric acid secretion | 2.94 | Mylk, Kcnk2, Atp1b1, Kcnk10, Plcb1, Kcnj10, Slc9a1, Mylk2, Gnaq, Slc9a4, Atp1a1, Plcb4, Kcnj1 |
| Circadian entrainment | 2.93 | Nos1, Rasd1, Rps6ka5, Plcb1, Cacna1c, Per1, Kcnj9, Mtnr1b, Cacna1d, Gnaq, Gng8, Gng7, Mtnr1a, Plcb4, Cacna1g |
| Inositol phosphate metabolism | 2.62 | Mtm1, Plcb1, Mtmr3, Plcz1, Synj2, Pik3c3, Inpp4b, Impa2, Plcd1, Plcb4, Pik3c2g, Plcg2 |
| GnRH signaling pathway | 2.34 | Mapk10, Map2k4, Egfr, Map2k6, Plcb1, Cacna1c, Cacna1d, Gnaq, Mmp2, Map3k4, Ptk2b, Pld1, Plcb4 |

The top 10 most significant path entries for enrichment are depicted.

**Table S3 KEGG pathway list for DhMRs showing hyper-5hmC in gene body regions of cardiac tissue of STZ vs control**

| **Pathway** | **-log10(FDR)** | **Gene list** |
| --- | --- | --- |
| Transcriptional misregulation in cancer | 2.71 | Flt1, Etv6, Ccna2, Smad1, Cdk9, Zbtb16, Hmga2 |
| Metabolic pathways | 2.47 | Man1a1, Atp6v1g2, Umps, Pfkfb2, Nt5c3a, Ptgs1, Csgalnact2, Nsd3, Pccb, Agpat4, Aldh1a7, Cds2, Ext1, Plcb4, Pde4d, Ugt2b15, Gdpd1 |
| Cellular senescence | 2.40 | Calm1, Ppp1cb, Hipk2, Ets1, Hipk3, Ccna2 |
| Long-term potentiation | 2.21 | Calm1, Ppp1cb, Camk4, Plcb4 |
| Adrenergic signaling in cardiomyocytes | 2.03 | Calm1, Ppp1cb, Crem, Cacna2d3, Plcb4 |
| Alcoholism | 2.03 | Hat1, Calm1, Ppp1cb, Gnb3, Camk4 |
| Oxytocin signaling pathway | 2.03 | Calm1, Ppp1cb, Camk4, Cacna2d3, Plcb4 |
| Calcium signaling pathway | 2.03 | Flt1, Calm1, Ppif, Trdn, Camk4, Plcb4 |
| Parathyroid hormone synthesis, secretion and action | 1.743 | Akap13, Jund, Plcb4, Pde4d |
| Pathways in cancer | 1.603 | Calm1, Ets1, Cul2, Ccna2, Gnb3, Zbtb16, Plcb4, Axin2 |

The top 10 most significant path entries for enrichment are depicted.

**Table S4. Physiological, cardiac, and serum parameters of experimental animals**

|  | **Control** | **STZ** | **Control + AKG** | **STZ + AKG** |
| --- | --- | --- | --- | --- |
| **Physiological parameters** | | | | |
| **Body weight (gms)** | 360  ±14.5 | 235.8  ±10.51**** | 371  ±9.402 ^ns^ | 252.3  ±8.969**** |
| **HW/BW ratio (mg/gm)** | 2.51  ±0.082 | 3.37  ±0.14*** | 2.54  ±0.067^ns^ | 3.03  ±0.14* |
| **Blood glucose levels (mg/dL)** | 100.5  ±5.78 | 488.8  ±11.66**** | 94.4  ±5.54^ns^ | 411.8  ±8.66****^####^ |
| **ECG parameters** | | | | |
| **Heart rate (BPM)** | 274.8  ±27.96 | 166.2  ±13.08** | 237.4  ±19.27 ^ns^ | 226.7  ±20.39^ns^ |
| **RR interval (ms)** | 241.9  ±23 | 352.7  ±29.81* | 260.5  ±22.46^ns^ | 276.2  ±27.27^ns^ |
| **QRS interval (ms)** | 17.71  ±0.39 | 19.62  ±0.63^ns^ | 17.85  ±0.63 ^ns^ | 15.22  ±0.55*^##^ |
| **QTc interval (ms)** | 149.4  ±19.52 | 171.1  ±7.23^ns^ | 166.8  ±8.39^ns^ | 156.1  ±11^ns^ |
| **Hemodynamic parameters** | | | | |
| **Maximum arterial pressure (mmHg)** | 125  ±3.4 | 151.7  ±6.504** | 118.5  ±2.9^ns^ | 133.4  ±4.54^ns^ |
| **Minimum arterial pressure (mmHg)** | 86.03  ±2.13 | 106.9  ±5.4* | 68.27  ±3.71^ns^ | 97.95  ±5.97^ns^ |
| **Diastolic duration (s)** | 0.15  ±0.011 | 0.24  ±0.0072*** | 0.16  ±0.018 ^ns^ | 0.15  ±0.021 ^ns ##^ |
| **Systolic duration (s)** | 0.091  ±0.0028 | 0.15  ±0.0041**** | 0.092  ±0.0057^ns^ | 0.12  ±0.0057**^###^ |
| **Maximum dp/dt (mmHg/s)** | 963.9  ±16.86 | 753.3  ±47.3* | 1136  ±41.34 ^ns^ | 628.5  ±58.62*** |
| **Minimum dp/dt (mmHg/s)** | -522.4  ±25.43 | -381.7 ±  16.35*** | -543.6 ±  25.52 ^ns^ | -335.5 ±  22.37**** |
| **Tau (s)** | 0.06878 ±0.0045 | 0.2221 ±0.015**** | 0.09475 ±0.0093 ^ns^ | 0.1292  ±0.020*^###^ |
| **Serum parameters** | | | | |
| **Insulin ng/ml** | 0.58  ±0.04 | 0.26  ±0.03** | 0.63  ±0.05^ns^ | 0.34  ±0.05* |
| **CPK U/L** | 271.6  ±15.13 | 332.6  ±14.07* | 283.6  ±11.97^ns^ | 296.7  ±7.17^ns^ |
| **CK-MB** **U/L** | 31.5  ±1.82 | 38.52  ±3.01^ns^ | 29.09  ±1.33^ns^ | 33.55  ±1.78^ns^ |
| **Total cholesterol (mg/dL)** | 61.2  ±2.68 | 79  ±1.99** | 63.43  ±2.13 ^ns^ | 68.7  ±3.69^ns^ |
| **Triglycerides (mg/dL)** | 56.08  ±3.28 | 85.9  ±6.06** | 63.98  ±3.13^ns^ | 66.2  ±5.44^ns #^ |

HW/BW, the ratio of heart weight to body weight, ECG, electrocardiography, BPM, beats per minute, dp/dt, rate of change of pressure.

Values are expressed as the mean ± SEM, and significant differences are depicted as *p < 0.05, **p < 0.001, ***p < 0.0002, ****p < 0.0001 vs the control group.

#p < 0.05, ##p < 0.001, ###p < 0.0002, ####p < 0.0001 vs STZ group.

**Table S5** Primer and oligos sequences.

| **Sr.No.** | **Name of gene** | | **5ʹ to 3ʹ Sequence of primer** |
| --- | --- | --- | --- |
| 1 | DNMT1 qPCR | Forward | CCACCACCAAGCTGGTCTAT |
|  |  | Reverse | TACGGCCAAGTTAGGACACC |
| 2 | DNMT3A qPCR | Forward | TGGCAAATTCTCAGTGGTGTGTG |
|  |  | Reverse | TGGGCTGCTTGTTGTAGGTG |
| 3 | DNMT3B qPCR | Forward | CAACAACAAGCAACCAGAGGTTC |
|  |  | Reverse | GCGTCTCCTGGCTTCTAAGTTC |
| 4 | TET1 qPCR | Forward | TGTCACCTGTTGCATGGATT |
|  |  | Reverse | TTGGATCTTGGCTTTCATCC |
| 5 | TET2 qPCR | Forward | TCGGAGGAGAAGAGTCAGGA |
|  |  | Reverse | TAGGGCTTGCATTTTCCATC |
| 6 | TET3 qPCR | Forward | ACCCGGCTCTATGAAACCTT |
|  |  | Reverse | GGCTGCAGACTTGATCTTCC |
| 7 | NFATC1 qPCR | Forward | GCTACAGCCGCAGTAAATGAG |
|  |  | Reverse | CTTCTGCCAGCTCCAATGTG |
| 8 | MYH10 qPCR | Forward | CATTTGGAAATGCGAAGACTGTG |
|  |  | Reverse | CGTGCGTTCATCTTTAGCTTGAC |
| 9 | ATPAF2 qPCR | Forward | GCATGATGTGGCGAATCTACCC |
|  |  | Reverse | TTATCTCAAAGCCACCTTCACCC |
| 10 | RXRA qPCR | Forward | CTCGCTGTTGAGCCCAAGACTG |
|  |  | Reverse | AACAGGGTCATTTGGTGAGCTG |
| 11 | DUSP26 qPCR | Forward | CTTGCGACCAGGAGCAACA |
|  |  | Reverse | TCTTCAAGGTGGCGGGATCT |
| 12 | OGDH qPCR | Forward | AGCTGAACAGGAGACAGGTATTTG |
|  |  | Reverse | GTCTGGGAGGCTGTCAATGG |
| 13 | PLN qPCR | Forward | CTGAGCTCCCAGACTTCACAC |
|  |  | Reverse | GGCATTTCAATAGTCGAGGCTCTC |
| 14 | TGFBR2 qPCR | Forward | ACCTTCTTCATGTGCTCCTGTAAC |
|  |  | Reverse | AGGACTGCTGGTGGTGTATTCTTC |
| 15 | TGFBR3 qPCR | Forward | ACGAGCATGTTTATGTTGAGGTGTC |
|  |  | Reverse | AAGCAGGTTTGGATGGCGAATC |
| 16 | ACTB qPCR | Forward | GCAGATGTGGATCAGCAAGC |
|  |  | Reverse | AGAAAGGGTGTAAAACGCAGC |
| 17 | DUSP26 MEDIP-qPCR | Forward | ATACGCAAGGTACGCAAGGTG |
|  |  | Reverse | GCACTTACCACATGCTAGGCAC |
| 18 | ATPAF2 MEDIP-qPCR | Forward | ACACACACTCTACTGCTTCC |
|  |  | Reverse | AACTACAAGTCCCAGAGGGC |
| 19 | TGFBR2 MEDIP-qPCR/chip-PCR | Forward | ATATGGGGATTCGGGGCATACTTC |
|  |  | Reverse | AGGAAATTCTACCCAGAGCTGTCTC |
| 20 | OGDH hMEDIP-qPCR | Reverse | TGCCTTCTAATGACAGCCGAAAG |
|  |  | Forward | GCTTGTGGGCGTAACATGAC |
| 21 | PLN hMEDIP-qPCR | Reverse | GCAACTGTTCCCATAAACCTAGG |
|  |  | Forward | CCAAAGTCAGTGATACCGTGCC |
| 22 | TGFBR3 hMEDIP-qPCR/chip-PCR | Forward | GTGTGTACCTGTGCGTTAAAACAG |
|  |  | Reverse | TCAAGAGGAAGCATCTTTGAGCG |
| 23 | DNMT3B shRNA oligo sequence | Forward | CCGGCTGTTCAGCCAGCACTTTAATCTCGAGATTAAAGTGCTGGCTGAACAGTTTTTG |
|  |  | Reverse | AATTCAAAAACTGTTCAGCCAGCACTTTAATCTCGAGATTAAAGTGCTGGCTGAACAG |

**Fig. S1 GO enrichment analysis.** Significantly enriched GO terms in (A) MEDIP-seq and (B) hMEDIP-seq analysis. Red, green, and blue bars represent the biological process, cellular component, and molecular function respectively. The x-axis represents -log10(p-value), the y-axis represents GO terms and the number to the right of the column indicates the number of enriched genes.

**
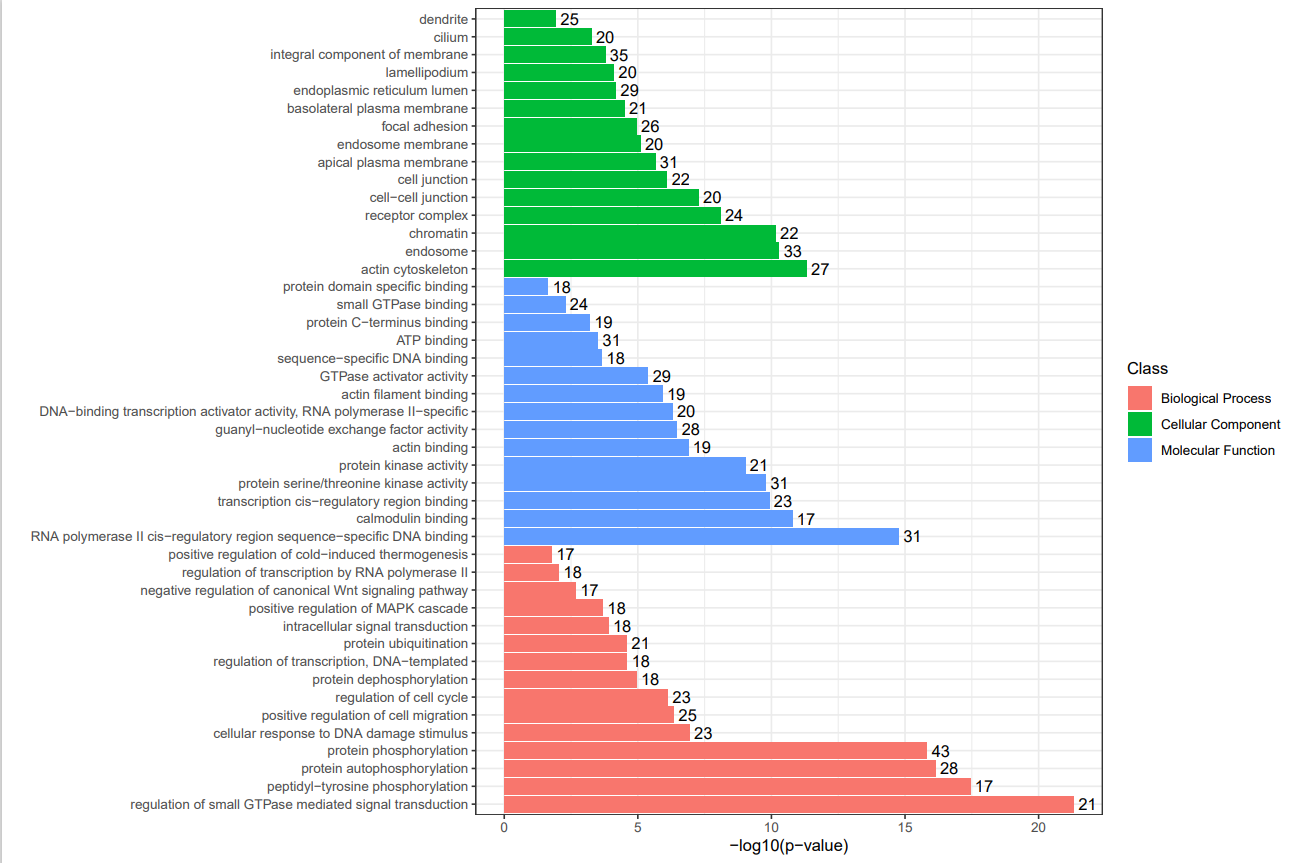
A)**

**B)**

**
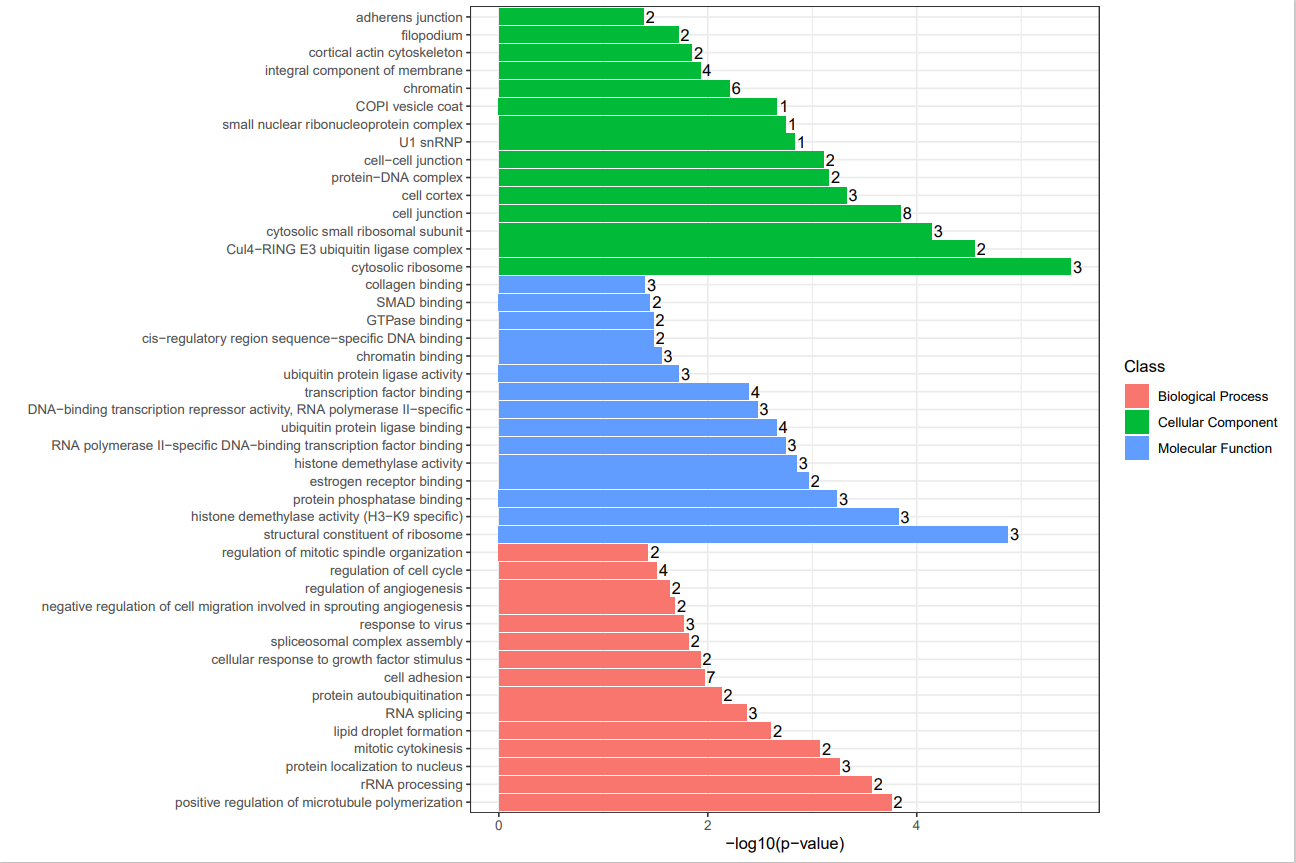
**

**Fig. S2 Enrichment of DMRs and DhMRs within gene body regions visualized by IGV.** Locations of the differentially methylated and hydroxymethylated peaks within gene body regions were visualized by IGV genome browser. Hypermethylated/ hyperhydroxymethylated peaks are denoted by black color and hypomethylated/ hypohydroxymethylated peaks are denoted by red color. Refseq gene is shown in the first track, hMEDIP peaks in the middle track, and MEDIP peaks are in the lower track.


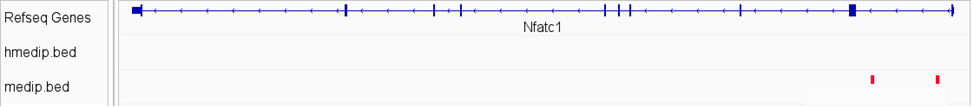

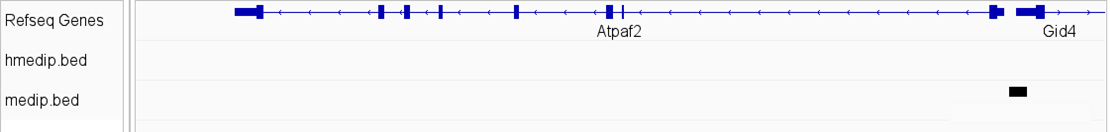

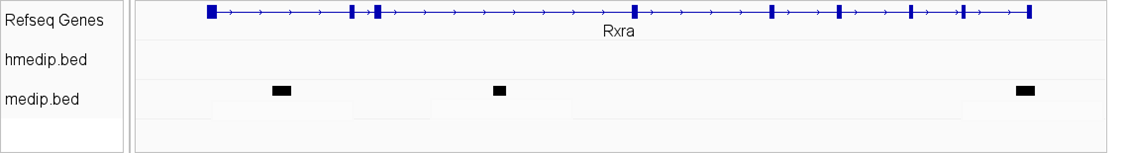

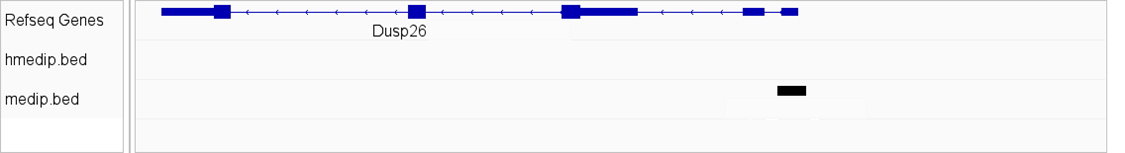

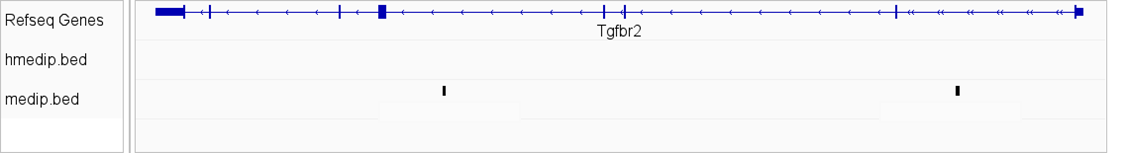

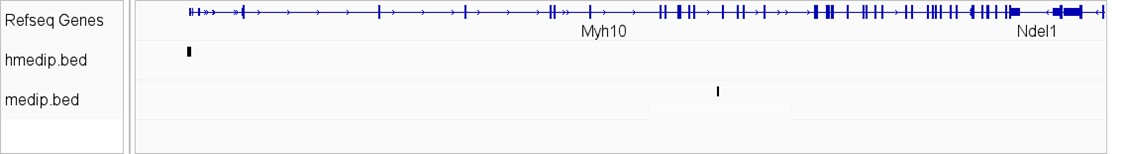

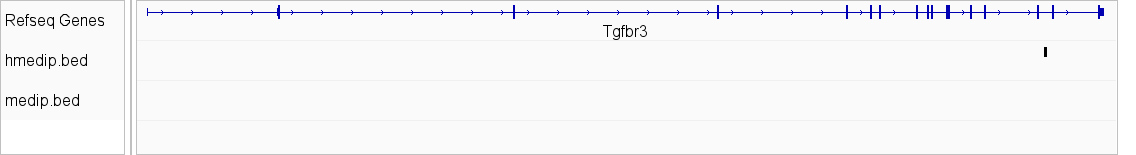

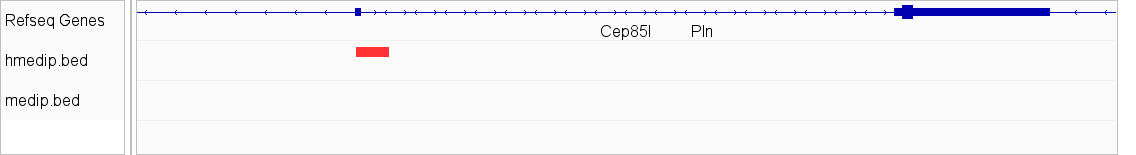

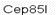

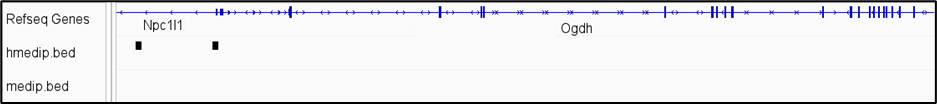


**Fig. S3 H&E staining micrographs.** H&E-stained cardiac sections from three different rat LV tissues from each group; arrows indicate lymphocyte infiltration, and arrowheads indicate degeneration of myocardial fibers.

**
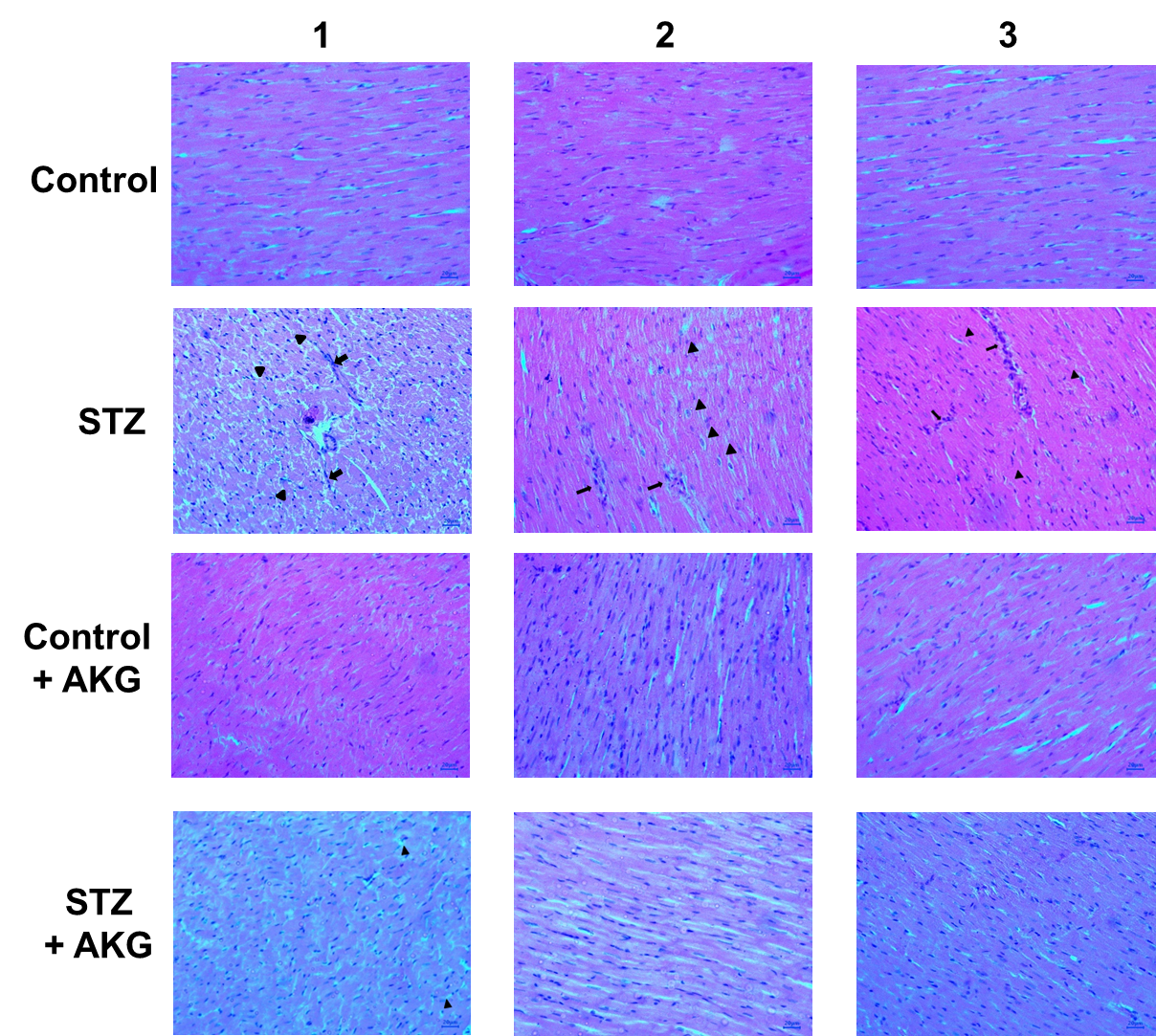
**

**Fig. S4 Densitometry analysis of western blot data.** Densitometry analysis of western blot data is shown in Fig. 5B for (A) DNMT1, (B) DNMT3A, (C) DNMT3B, (D) MeCP2, (E) MBD2, and (F) TET1. Band intensities are normalized to Vinculin and represented as mean ± SD for three different animals. Statistical significance was determined by one-way ANOVA with Tukey’s multiple comparisons post-test where, *p < 0.05, **p < 0.001, ****p < 0.0001vs control, and ###p < 0.0002 vs STZ by one-way ANOVA.

**
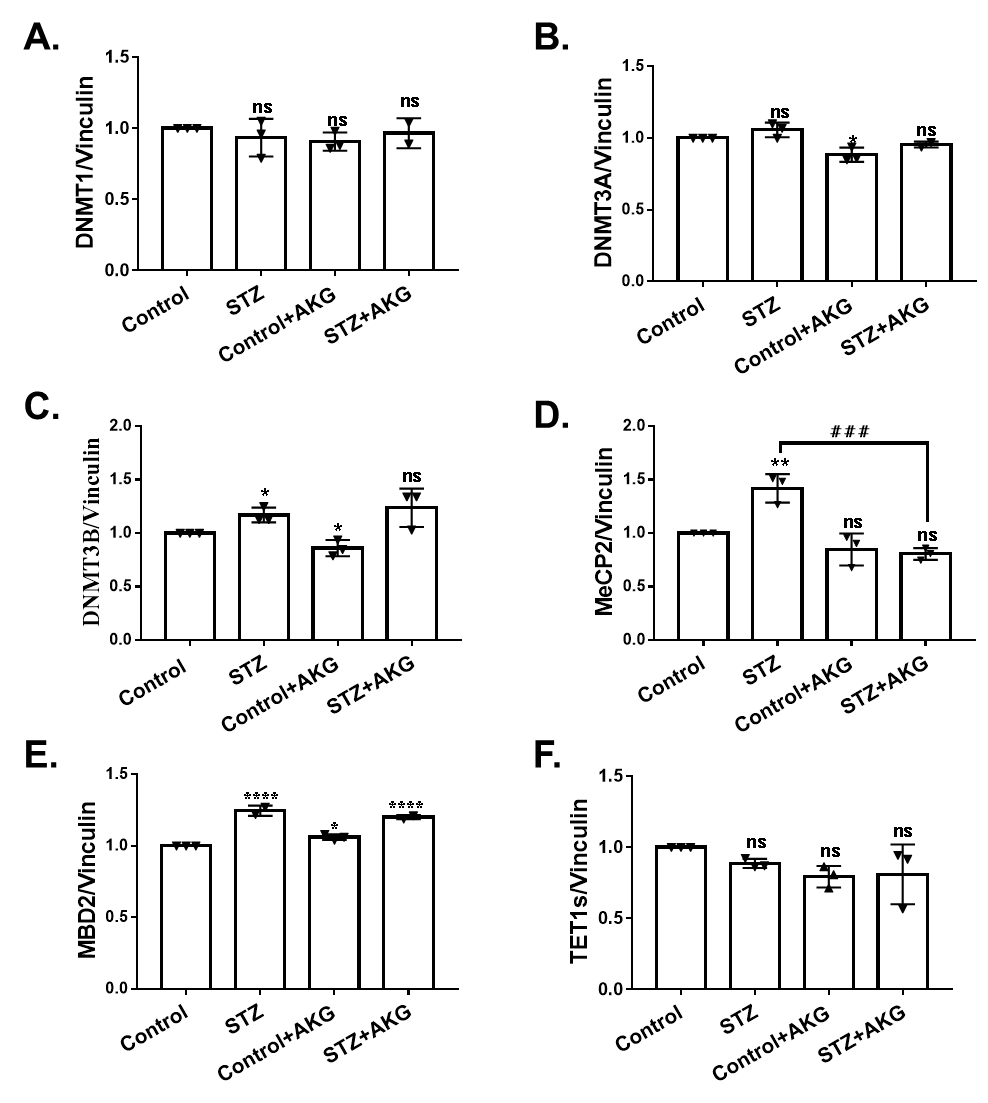
**

**Fig. S5 Quantitative analysis of global 5mC/5hmC levels in *in vitro* experiments. (A, B)** Densitometry analysis of 5mC- and 5hmC-specific dot blot analysis of gDNA isolated from NG- and HG-treated H9c2 cells in the presence of AKG treatment (A) 5mC levels and (B) 5hmC levels. **(C, D)** Densitometry analysis of 5mC- and 5hmC-specific dot blot analysis of gDNA isolated from NG- and HG-treated DNMT3b kockdown-H9c2 cells (C) 5mC levels and (D) dot blot intensities are normalized to methylene blue staining. (E,F) Immunofluorescence intensities of 5mC antibody staining in presence of NG or HG in (E) AKG-supplemented and (F) DNMT3b knockdown H9c2 cells fluorescence intensities were normalised to the DAPI staining. Results are represented as mean ± SD for three individual experiments and statistical significance was determined by one-way ANOVA with Tukey’s multiple comparisons post-test where, **p < 0.001, ***p < 0.0002, ****p < 0.0001vs control, and ##p < 0.001, ###p < 0.0002, ####p < 0.0001 vs STZ.


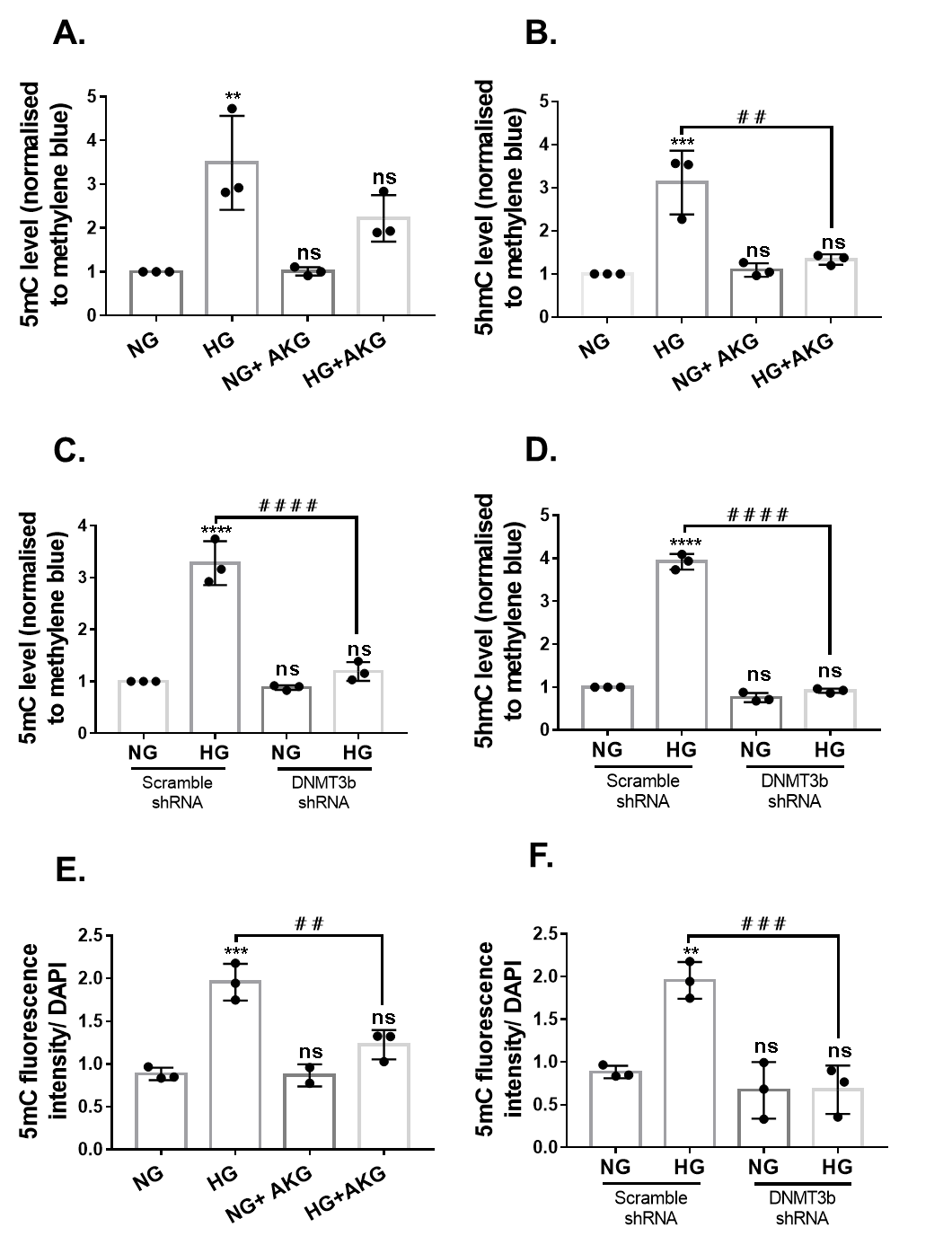

Supplement: Supplementary file 1 — Additional file 1: Table S1. KEGG pathway list for DMRs showing hypo-5mC in gene body regions of cardiac tissue of STZ vs control. Table S2. KEGG pathway list for DMRs showing hyper-5mC in gene body regions of cardiac tissue of STZ vs control. Table S3. KEGG pathway list for DhMRs showing hyper-5hmC in gene body regions of cardiac tissue of STZ vs control. Table S4. Physiological, cardiac, and serum parameters of experimental animals. Table S5. Primer and oligos sequences. Fig. S1. GO enrichment analysis. Significantly enriched GO terms in (A) MEDIP-seq and (B) hMEDIP-seq analysis. Red, green, and blue bars represent the biological process, cellular component, and molecular function respectively. The x-axis represents -log10(p-value), the y-axis represents GO terms and the number to the right of the column indicates the number of enriched genes. Fig. S2. Enrichment of DMRs and DhMRs within gene body regions visualized by IGV. Locations of the differentially methylated and hydroxymethylated peaks within gene body regions were visualized by IGV genome browser. Hypermethylated/ hyperhydroxymethylated peaks are denoted by black color and hypomethylated/ hypohydroxymethylated peaks are denoted by red color. Refseq gene is shown in the first track, hMEDIP peaks in the middle track, and MEDIP peaks are in the lower track. Fig. S3. H&E staining micrographs. H&E-stained cardiac sections from three different rat LV tissues from each group; arrows indicate lymphocyte infiltration, and arrowheads indicate degeneration of myocardial fibers. Fig. S4. Densitometry analysis of western blot data. Densitometry analysis of western blot data is shown in Fig. 5B for (A) DNMT1, (B) DNMT3A, (C) DNMT3B, (D) MeCP2, (E) MBD2, and (F) TET1. Band intensities are normalized to Vinculin and represented as mean ± SD for three different animals. Statistical significance was determined by one-way ANOVA with Tukey’s multiple comparisons post-test where, *p < 0.05, **p < 0.001, ****p < 0.0001vs contr [file 13072_2023_489_MOESM1_ESM.docx]
